# FAM172A promotes follicular thyroid carcinogenesis and may be a marker of FTC

**DOI:** 10.1530/ERC-20-0181

**Published:** 2020-09-21

**Authors:** Pei-Pei Xu, Su Zeng, Xiao-Tian Xia, Zi-Heng Ye, Mei-Fang Li, Ming-Yun Chen, Tian Xia, Jing-Jing Xu, Qiong Jiao, Liang Liu, Lian-Xi Li, Ming-Gao Guo

**Affiliations:** 1 Department of Surgery, Shanghai Jiao Tong University Affiliated Sixth People’s Hospital, Shanghai, China; 2 Department of Emergency, Shanghai Jiao Tong University Affiliated Sixth People’s Hospital, Shanghai, China; 3 Department of Endocrinology and Metabolism, Shanghai Jiao Tong University Affiliated Sixth People’s Hospital, Shanghai Clinical Center for Diabetes, Shanghai, China; 4 CAS Key Laboratory of Molecular Virology and Immunology, Institute Pasteur of Shanghai, Chinese Academy of Sciences, Shanghai, China; 5 Department of Pathology, Shanghai Public Health Clinical Center, Fudan University, Shanghai, China; 6 Department of Pathology, Shanghai Jiao Tong University Affiliated Sixth People’s Hospital, Shanghai, China

**Keywords:** follicular thyroid carcinoma, follicular thyroid adenoma, FAM172A, fine-needle aspiration biopsy

## Abstract

Our aims were to uncover the role of FAM172A (Family with sequence similarity 172 member A) in the pathogenesis of follicular thyroid carcinoma (FTC) and to evaluate its value in the differential diagnosis between malignant and benign thyroid follicular lesions. FAM172A expression was evaluated by q-PCR, immunoblotting and immunohistochemistry (IHC). The ability of proliferation, migration and invasion of cells were assessed by Cell Counting Kit-8 assay (CCK8), clone-formation and Transwell assays. Nude mouse tumorigenicity assays were used to investigate the role of FAM172A in the pathogenesis of FTC *in vivo*. The value of FAM172A in the differential diagnosis for FTC was assessed using 120 formalin-fixed paraffin-embedded (FFPE) tissues after the operation and 81 fine-needle aspiration biopsy (FNAB) samples before the operation. FAM172A was highly expressed in FTC tissues and FTC cell lines. Downregulation of FAM172A inhibited the proliferation, invasion and migration of FTC cells through Erk1/2 and JNK pathways. Subcutaneous tumorigenesis in nude mice showed that knockdown of FAM172A inhibited tumor growth and progression *in vivo*. The FAM172A IHC scores of 3.5 had 92% sensitivity and 63% specificity to separate FTC from benign/borderline thyroid follicular lesions, and 92% sensitivity and 80% specificity to discriminate FTC from benign thyroid follicular lesions in postoperative FFPE samples. The corresponding values were 75 and 78%, and 75 and 89% in preoperative FNA samples, respectively. FAM172A plays an important role in the pathogenesis of FTC through Erk1/2 and JNK pathways. FAM172A may be a potential marker for the preoperative diagnosis of FTC based on the IHC results of thyroid FNAB samples.

## Introduction

Thyroid carcinoma includes papillary thyroid carcinoma (PTC), follicular thyroid carcinoma (FTC), medullary thyroid carcinoma (MTC) and anaplastic thyroid carcinoma (ATC) (Bychkov 2017). Among them, FTC has unique biological behaviors, such as distant metastasis in the early stage of cancer, and less favorable outcomes compared with PTC (Grani *et al.* 2018). The incidence of FTC has declined in the past few years, but still is the second most common thyroid malignancy, accounting for 10–15% of all thyroid cancers (Siegel *et al.* 2018).

Given the poor prognosis after distant metastasis, such as lung and bone metastases, it is crucial for early and precise diagnosis and timely treatment of FTC based on characteristic clinical manifestations. However, it is difficult to identify FTC only through clinical manifestations, ultrasonography and cytology before operation (Cipriani *et al.* 2015). Currently, fine-needle aspiration biopsy (FNAB) cytology is the gold standard for diagnosing other thyroid cancers such as PTC, MTC and ATC, but only capsular and vascular invasion can be used to differentiate FTC from benign follicular thyroid lesions based on postoperative pathology (Cipriani *et al.* 2015), which indicates that FNAB is unable to distinguish malignant from benign thyroid follicular lesions (Mazzanti *et al.* 2004, Cooper *et al.* 2009, Layfield *et al.* 2009, Olson *et al.* 2012).

Therefore, the search for specific markers of FTC remains one of the most important issues in distinguishing malignant from benign thyroid follicular lesions. Currently, several immunohistochemistry (IHC) markers have been suggested to improve the diagnostic accuracy of thyroid follicular epithelial cancer (Bartolazzi *et al.* 2001, Goldstein *et al.* 2002, Gustafson *et al.* 2003, Cochand-Priollet *et al.* 2011). For example, IHC staining for Hector Battifora mesothelial antigen-1 (HBME-1) was useful to pick out malignant thyroid tumors from indeterminate thyroid follicular tumors (Cochand-Priollet *et al.* 2011).

On the other hand, the pathogenetic mechanisms of FTC remains to be fully elucidated compared with PTC. The genetic alterations, such as gene mutations, deletions and translocations can simultaneously activate the signaling pathways related to thyroid cancers (Xing 2013), but the mechanisms underlying this process remain poorly understood. Therefore, the pathways and protein molecules involved in the development and progression of FTC also need further research and clarification.

FAM172A is a novel protein and was confirmed for the first time in the world by our group (Li *et al.* 2010). Recently, a study by our group found that FAM172A is highly expressed in PTC tissues and promotes PTC progression (Li *et al.* 2016). Based on these findings, we hypothesized that FAM172A may also play an important role in the pathogenesis of FTC and may be used as a potential diagnostic marker for FTC. Therefore, one of the aims of the present study is to explore the role and mechanism of FAM172A in the pathogenesis of FTC. Another aim is to assess the value of FAM172A in the preoperative diagnosis of FTC.

## Materials and methods

### Case selection

Human FTC tumor tissues and adjacent non-tumor tissues were collected from six patients with confirmed FTC. 120 FFPE samples and 81 patients underwent FNAB were obtained. The study was approved by the ethics committee of Shanghai Jiao Tong University Affiliated Sixth People’s Hospital, and written informed consent was obtained from all patients. Further details of case collection can be found in the Supplementary Materials and methods (see section on supplementary materials given at the end of this article).

### IHC analysis

The expression levels of FAM172A were assessed by IHC analysis. Based on the percentage and intensity of the positively stained tumor cells under microscopy (400×), a semi-quantitative scoring system was applied to assess the staining results (Bovee *et al.* 2006, Douwes Dekker *et al.* 2007, Hinkel *et al.* 2008, Pansuriya *et al.* 2011, Lai *et al.* 2017). Finally, the percentage score × the intensity score ≥ 3.5 was considered as a standard to prospectively predict the presence of FTC in FNAB samples. Further details of IHC analysis are presented in the Supplementary Materials and methods.

### Cell culture

All cell lines including Nthy-ori 3-1, FTC-133 and FTC-238 were maintained in RPMI 1640 medium (Gibco) supplemented with 10% fetal bovine serum (FBS, Gibco). Cells were maintained at 37°C in a humidified 5% CO2 incubator. All cell lines were authenticated using short tandem repeat (STR) analysis. Further details of cell culture are presented in the Supplementary Materials and methods.

### RNA extraction and RT-PCR

RNA extraction and RT-PCR were performed according to a standard protocol, as detailed in the Supplementary Materials and methods.

### Immunoblotting

Immunoblotting was performed according to a standard protocol as detailed in the Supplementary Materials and methods.

### Establishment of stable cell lines of FAM172A overexpression and downregulation

Human FAM172A gene sequence was cloned into lentiviral vector PDS159_pL6.3-CMV-GFPa1-IRES-MCS to generate the PDS159-FAM172A plasmid. Human FAM172A specific shRNA (TRC shRNA TRCN0000127701) and control shRNA (TRC shRNA TRCN0000072223) were constructed according to pLKO.1 Protocol (Root *et al.* 2006). Further details are presented in the Supplementary Materials and methods.

### Cell proliferation and colony formation assays

Cell Counting Kit-8 (CCK8) (Biotool, Houston, TX, USA) was used as the end point to quantitatively assess the proliferation ability of cells. The soft agar colony formation assay was applied to evaluate the ability of a single cell to grow into a colony. Further details of cell proliferation and colony formation assays are presented in the Supplementary Materials and methods.

### Transwell assay

Transwell assay was performed to access the ability of migration and invasion. Further details of Transwell assay are presented in the Supplementary Materials and methods.

### Tumorigenicity assay *in vivo*

Animal care and experiments were approved by the committee for human treatment of animals at Shanghai Jiao Tong University Affiliated Sixth People’s Hospital. Further details of tumorigenicity assay are presented in the Supplementary Materials and methods.

### Next-generation sequencing technology

The next-generation sequencing work was performed by Amplicon-gene Bioscience (Shanghai, China) (https://www.amplicongene.com). Further details of next-generation sequencing are presented in the Supplementary Materials and methods.

### The signaling pathways associated with the role of FAM172A in FTC

The mechanisms of FAM172A involved in MAPK pathways were further investigated. The expression of p-JNK, JNK, p-Erk1/2 and Erk1/2 were detected by immunoblotting. Additionally, JNK inhibitor JNK-IN-7 and Erk1/2 inhibitor ravoxertinib were also used to further explore the role and mechanism of FAM172A in FTC. Further details are presented in the Supplementary Materials and methods.

### Statistical analysis

Where applicable, data were presented as the mean ± s.e.m. from at least three replicates. All independent experiments were performed in triplicate. Further details of statistical analysis are presented in the Supplementary Materials and methods.

## Results

### Upregulated expression of FAM172A in human FTC tissues and cell lines

**Figure 1:**
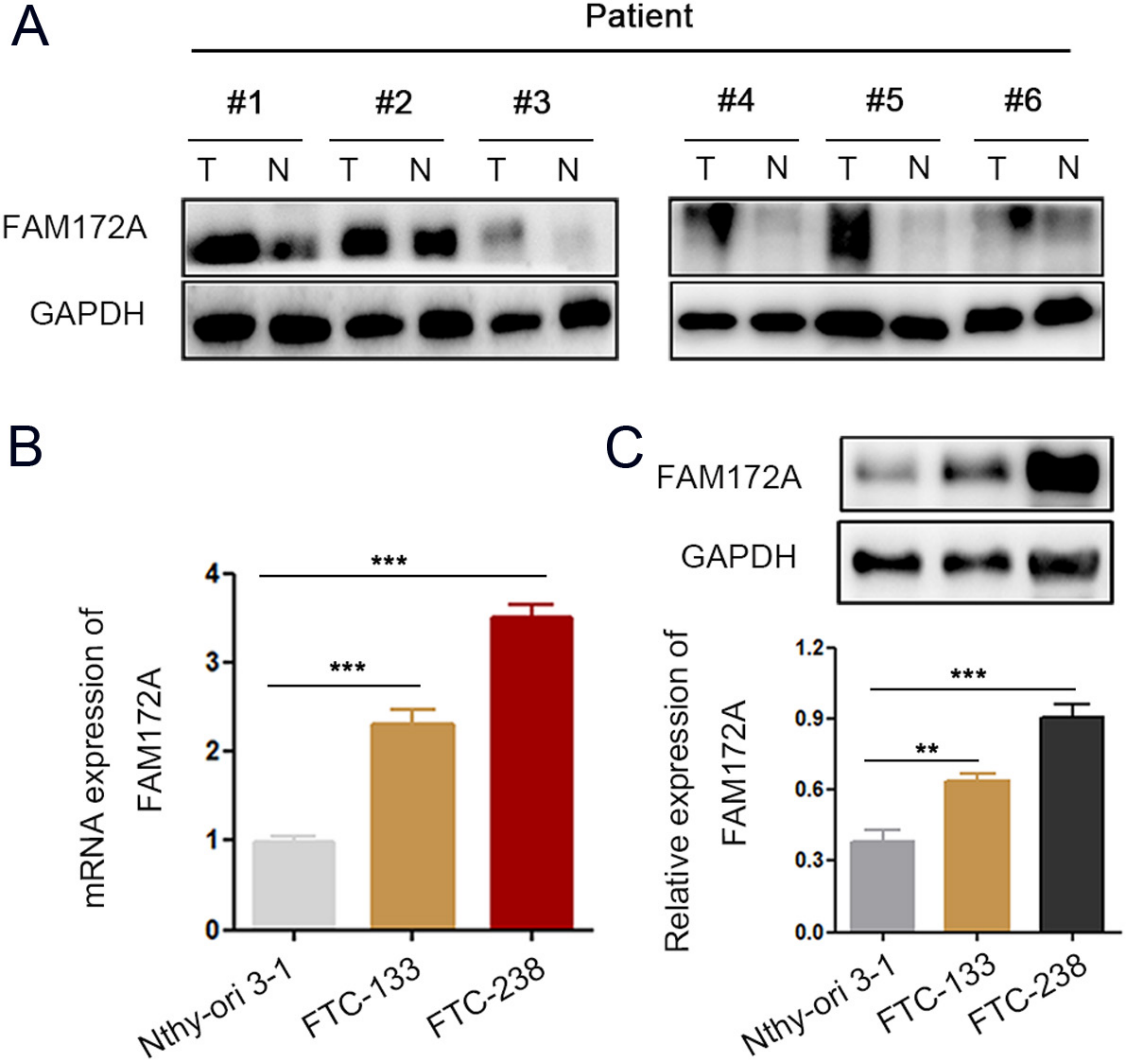
Upregulated expression of FAM172A in human FTC tissues and cell lines. (A) The comparison of immunoblotting results of FAM172A between FTC and peri-carcinoma tissues (*n* = 6). (B) The comparison of q-PCR results of FAM172A among Nthy-ori 3-1, FTC-133 and FTC-238 cells. (C) The comparison of immunoblotting results of FAM172A among Nthy-ori 3-1, FTC-133 and FTC-238 cells. (Differences between the two groups were analyzed using the independent t-test, values are expressed as the means ± s.e.m. **P < 0.01 and ***P < 0.001). A full color version of this figure is available at https://doi.org/10.1530/ERC-20-0181.

To determine the expression levels of FAM172A in FTC, Immunoblotting and q-PCR were performed in FTC tissues and cell lines. Immunoblotting results showed that the expression of FAM172A was obviously higher in FTC tissues than in peri-carcinoma tissues (Fig. 1A). Meanwhile, we also compared the expression levels of FAM172A in normal human thyroid follicular epithelial cell line Nthy-ori 3-1 and FTC cell line FTC-133 and FTC-238 by q-PCR and Immunoblotting. The results demonstrated that the expression of FAM172A was also significantly increased in FTC-133 and FTC-238 cells compared with normal thyrocytes Nthy-ori 3-1 (Fig. 1B and C). FAM172A antibody used in the study can recognize isoform1 and isoform2 of FAM172A. But the expression of FAM172A in thyroid cell lines is mainly expressed as isoform 1. Therefore, the role of isoform1 of FAM172A in FTC was intensively explored in the present study (Supplementary Fig. 1A).

### The effect of FAM172A on cell proliferation *in vitro*

**Figure 2:**
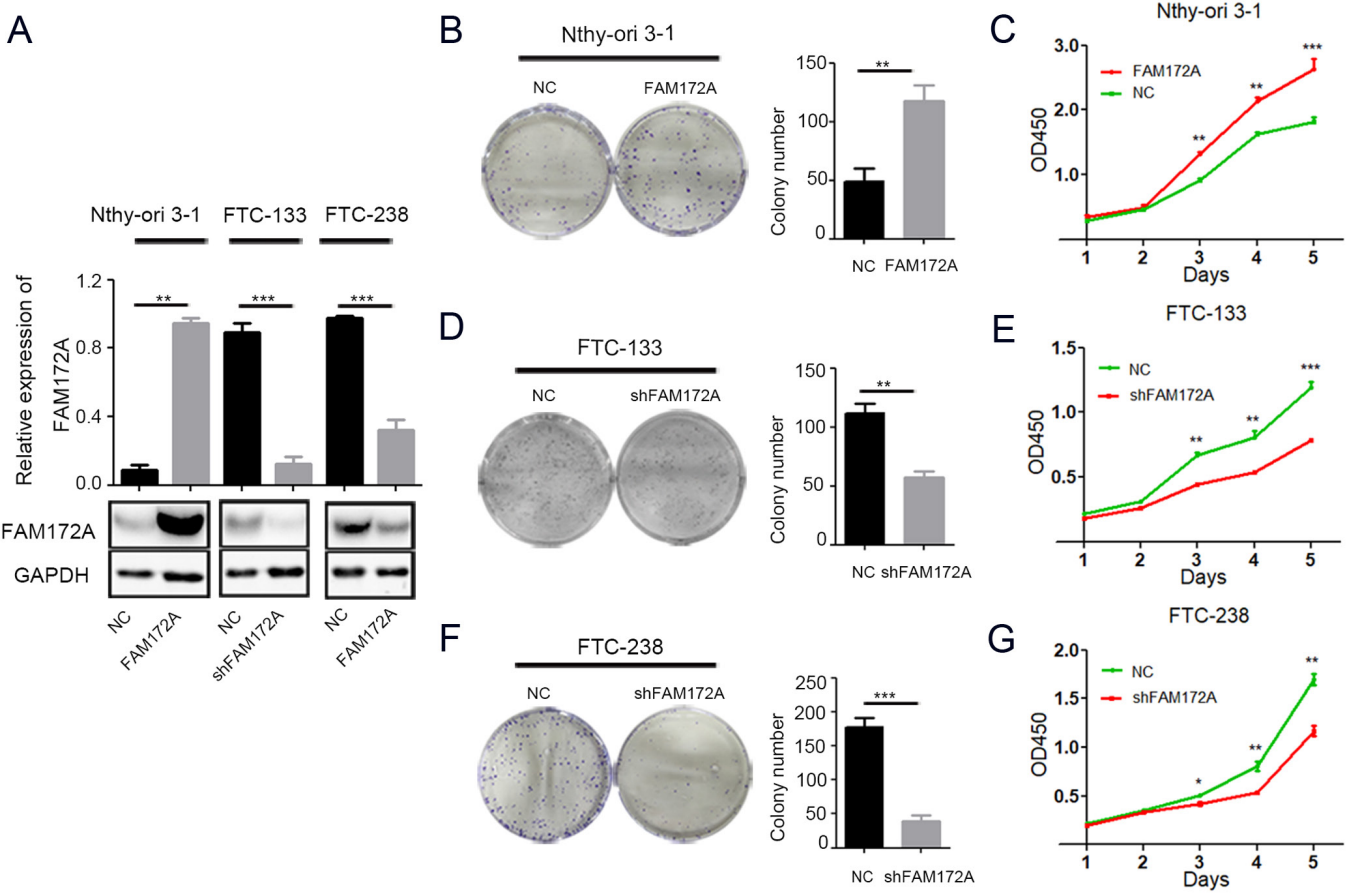
The effect of FAM172A on cell proliferation. (A) The efficiency of knockdown and overexpression of FAM172A gene validated by Western blotting. (B) The influence of FAM172A overexpression on colony-formation ability of Nthy-ori 3-1 cells. (C) The influence of FAM172A overexpression on the proliferation of Nthy-ori 3-1 cells determined by CCK-8 assay. (D) The influence of FAM172A knockdown on colony-formation ability of FTC-133 cells. (E) The influence of FAM172A knockdown on the proliferation of FTC-133 cells determined by CCK-8 assay. (F) The influence of FAM172A knockdown on colony-formation ability of FTC-238 cells. (G) The influence of FAM172A knockdown on the proliferation of FTC-238 cells determined by CCK-8 assay. (Differences between two groups were analyzed using the independent t-test, values are expressed as the means ± s.e.m. *P < 0.05, **P < 0.01 and ***P < 0.001). A full color version of this figure is available at https://doi.org/10.1530/ERC-20-0181.

We constructed a lentivirus vector pLKO.1-shRNA-FAM172A and established two stable knockdown cell lines using FTC-133 and FTC-238 cells. Meanwhile, we stably overexpressed FAM172A in normal human thyrocytes Nthy-ori 3-1 with lentivirus vector PDS159-FAM172A (Fig. 2A). The knockdown and overexpression of FAM172A gene were targeted the sequence of isoform1. The transduction efficiency of lentivirus vectors was verified by immunoblotting, which is shown in Fig. 2A. The knockdown efficiency was approximately 95% in FTC-133 and 80% in FTC-238 (Fig. 2A), respectively.

The effect of FAM172A on cell proliferation was investigated by colony-formation assay and CCK8 assay. The colony-formation assay showed that the overexpression of FAM172A in Nthy-ori 3-1 cells exhibited a significantly more and larger colonies than negative control NC-transfected cells (*P* < 0.01) (Fig. 2B), whereas downregulation of FAM172A in both FTC-133 and FTC-238 cells showed obviously fewer and smaller colonies than NC-transfected cells (*P* < 0.01 and *P* < 0.001, respectively) (Fig. 2D and F). Likewise, the CCK-8 assay demonstrated that FAM172A overexpression significantly promoted the proliferation of Nthy-ori 3-1 cells (*P* < 0.001) (Fig. 2C), whereas FAM172A downregulation clearly suppressed the proliferation of both FTC-133 and FTC-238 cells (*P* < 0.001 and *P* < 0.01, respectively) (Fig. 2E and G).

### The effect of FAM172A on cell migration and invasion *in vitro*

**Figure 3:**
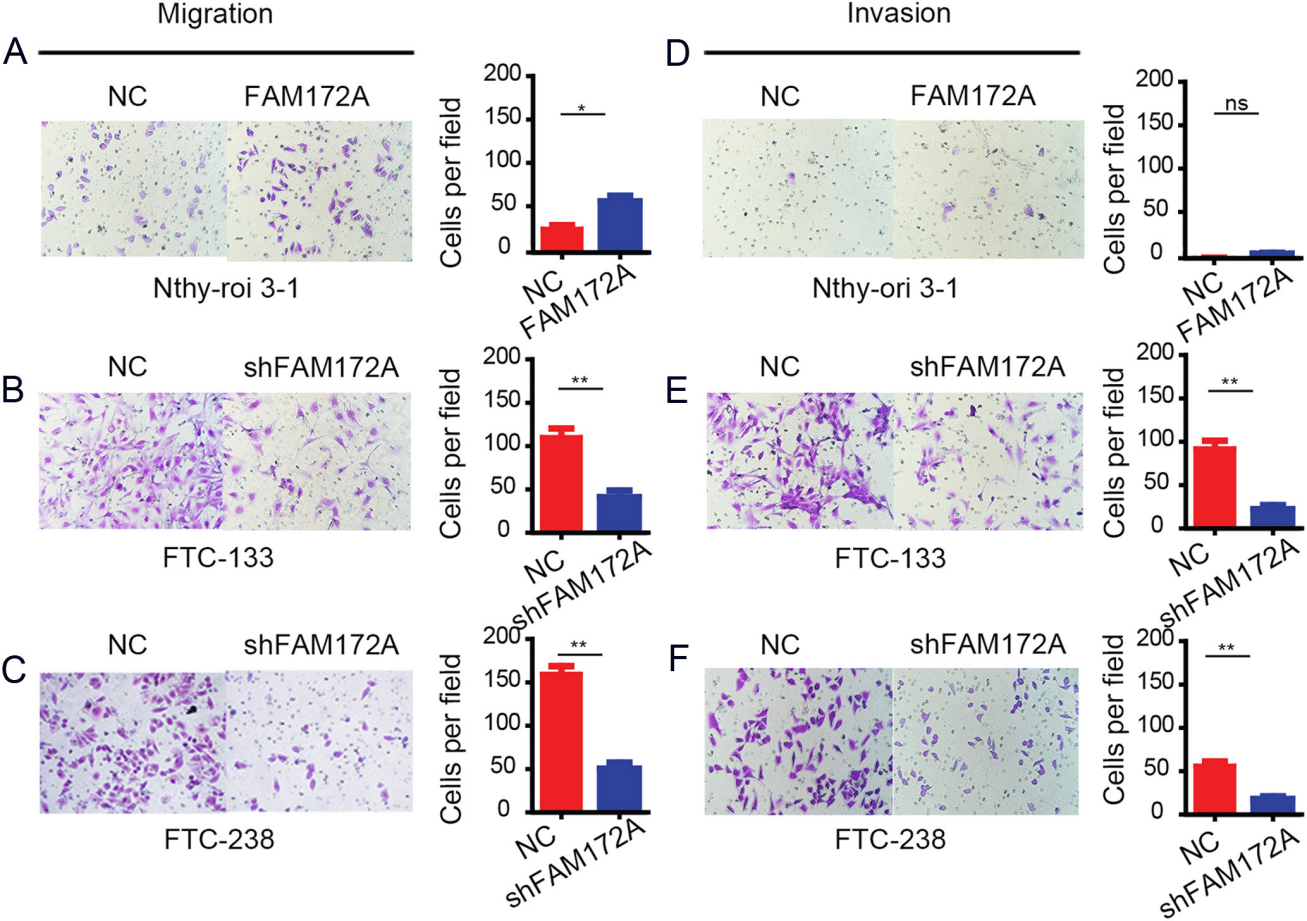
The effect of FAM172A on cell migration and invasion. (A) The influence of FAM172A overexpression on the migration of Nthy-ori 3-1 cells. (B) The influence of FAM172A knockdown on the migration of FTC-133 cells. (C) The influence of FAM172A knockdown on the migration of FTC-238 cells. (D) The influence of FAM172A overexpression on the invasion of Nthy-ori 3-1 cells. (E) The influence of FAM172A knockdown on the invasion of FTC-133 cells. (F) The influence of FAM172A knockdown on invasion of FTC-238 cells. (Differences between two groups were analyzed using the independent t-test or Mann–Whitney, values are expressed as the means ± s.e.m. *P < 0.05 and **P < 0.01).

Transwell assay was used to assess the effect of FAM172A on cell migration and invasion *in vitro*. The Transwell assay demonstrated that the overexpression of FAM172A significantly increased the number of migratory cells (by about 70%), but not the number of invasive cells compared with those transfected with control vectors in Nthy-ori 3-1 cells (Fig. 3A and D). Contrarily, the downregulation of FAM172A in both FTC-133 and FTC-238 cells obviously reduced the number of migratory and invasive cells, about reduced by 61 and 80% in FTC-133 (Fig. 3B and E) and 71 and 59% in FTC-238 cells (Fig. 3C and F), respectively.

### Knockdown of FAM172A suppresses tumorigenicity *in vivo*

**Figure 4:**
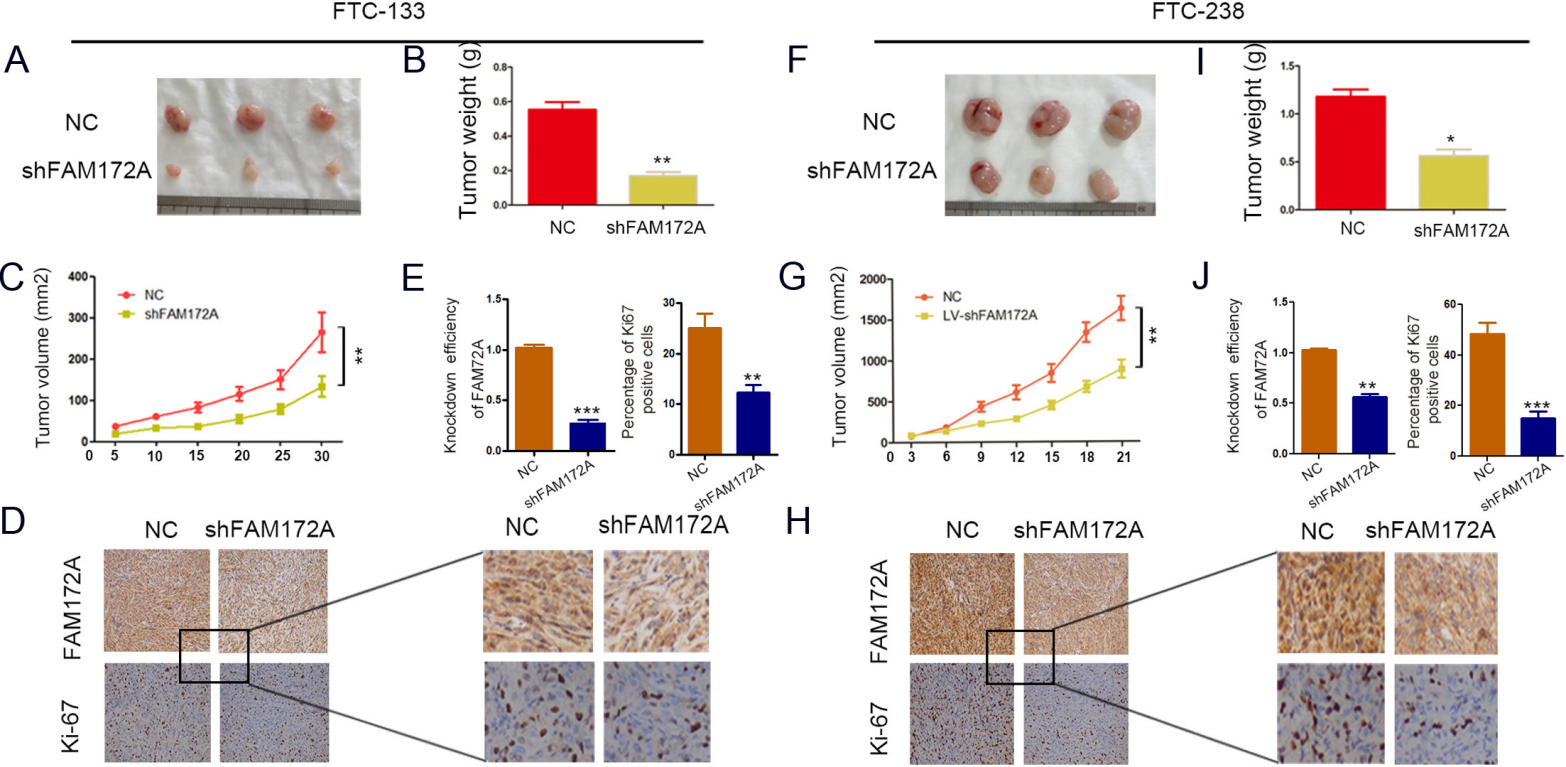
The influence of FAM172A knockdown on tumorigenicity of FTC cell lines. (A) The comparison of tumor morphology between the nude mice bearing FTC-133 cell with FAM172A knockdown and the negative control (*n* = 10). (B) The comparison of tumor weight between FAM172A knockdown-group and control group. (C) The comparison of tumor volume between FAM172A knockdown-group and control group. (D) The comparison of IHC of FAM172A and Ki-67 between FAM172A knockdown-group and control group. (E) The comparison of IHC semi-quantitative analyses of FAM172A and Ki-67 between FAM172A knockdown-group and control group (values are expressed as the means ± s.e.m. **P < 0.01 and ***P < 0.001). (F) The comparison of tumor morphology between the nude mice bearing FTC-238 cell with FAM172A knockdown and the negative control (*n* = 10). (G) The comparison of tumor weight between FAM172A knockdown-group and control group. (I) The comparison of tumor volume between FAM172A knockdown-group and control group. (H) The comparison of IHC of FAM172A and Ki-67 between FAM172A knockdown-group and control group. (J) The comparison of IHC semi-quantitative analyses of FAM172A and Ki-67 between FAM172A knockdown-group and control group. (Differences between two groups were analyzed using the independent t-test, values are expressed as the means ± s.e.m. *P < 0.05, **P < 0.01 and ***P < 0.001).

Based on the results of functional studies of FAM172A *in vitro*, FTC xenografts were established to investigate the effect of FAM172A on tumorigenesis *in vivo*. The results showed that the nude mice subcutaneously injected with shRNA-FAM172A-infected FTC-133 cells had significantly smaller tumors than those injected with NC-transfected FTC-133 cells (Fig. 4A). Both tumor weight (0.57 ± 0.14 g vs 0.16 ± 0.07 g, *P* < 0.01) and volume (295.57 ± 127.42 mm^3^ vs 149.09 ± 65.91 mm^3^, *P* < 0.01) were dramatically decreased in the nude mice injected with shRNA-FAM172A-infected FTC-133 cells compared with those injected with NC-transfected FTC-133 cells (Fig. 4B and C).

Similarly, the subcutaneous tumors formed from FTC-238 cells infected with shRNA-FAM172A lentivirus vector in nude mice were also markedly smaller than those formed from FTC-238 cells infected with empty vector (Fig. 4F). As expected, both tumor weight (1.18 ± 0.23 g vs 0.56 ± 0.22 g, *P* < 0.05) and volume (1626.62 ± 471.52 vs 885.18 ± 347.32 mm^3^, *P* < 0.01) were also obviously decreased in the nude mice injected with shRNA-FAM172A-infected FTC-238 cells when compared with the control group (Fig. 4I and G).

Furthermore, IHC staining of FAM172A and Ki-67 was performed in FTC xenograft tissues. The results showed that the expression of FAM172A was significantly decreased in the FAM172A-knockdowned FTC-133 (*P* < 0.001) and FTC-238 xenograft tissues (*P* < 0.01) (Fig. 4D, E, H and J). IHC staining of Ki-67 was used to assess the proliferative index of tumors. The results revealed that the expression levels of Ki-67 protein were clearly decreased in the FAM172A-knockdowned FTC-133 (*P* < 0.01) and FTC-238 (*P* < 0.01) xenograft tissues compared with control xenograft tissues (Fig. 4D, E, H and J). Given that Ki-67 is a well-known proliferation marker for the evaluation of cell proliferation, we performed Ki-67 IHC in 15 FTC samples to study the relationship between the expression level of FAM172A and Ki-67. The results showed that FAM172A expression was positively correlated with proliferation index Ki-67 (Supplementary Fig. 1B).

### FAM172A plays an important role in FTC through Erk1/2 and JNK MAPK pathways

To further clarify the pathogenic mechanisms of FAM172A in FTC, next-generation RNA-seq was performed between Nthy-ori 3-1 with empty vector and Nthy-ori 3-1 with lentivirus vector PDS159-FAM172A, and between FTC and peri-carcinoma tissue samples. The differentially expressed genes between the Nthy-ori 3-1 cell with and without overexpression of FAM172A were presented as Supplementary Fig. 2A. Likewise, the differentially expressed genes between FTC and peri-carcinoma tissues were also presented as Supplementary Fig. 2B. Finally, 61 overlapping genes were obtained by compared differentially expressed genes between two groups and were present as Supplementary Fig. 2C. Furthermore, five potentially important genes related to FTC were verified by qPCR in FTC cell lines with knockdown and overexpression of FAM172A and were present as Supplementary Fig. 2D.

Based on the results of next-generation RNA-seq, MAPK signaling pathway was chosen to intensively study. The results are presented in Supplementary Fig. 3. Differential expression genes were defined as P values less than 0.05 and were marked red. Some tumor-related genes such as FOX, NFATC1 and JUN were highly expressed in Nthy-ori 3-1 with lentivirus vector PDS159-FAM172A and FTC tissues compared with Nthy-ori 3-1 with empty vector and peri-carcinoma tissues (Supplementary Fig. 3).

**Figure 5:**
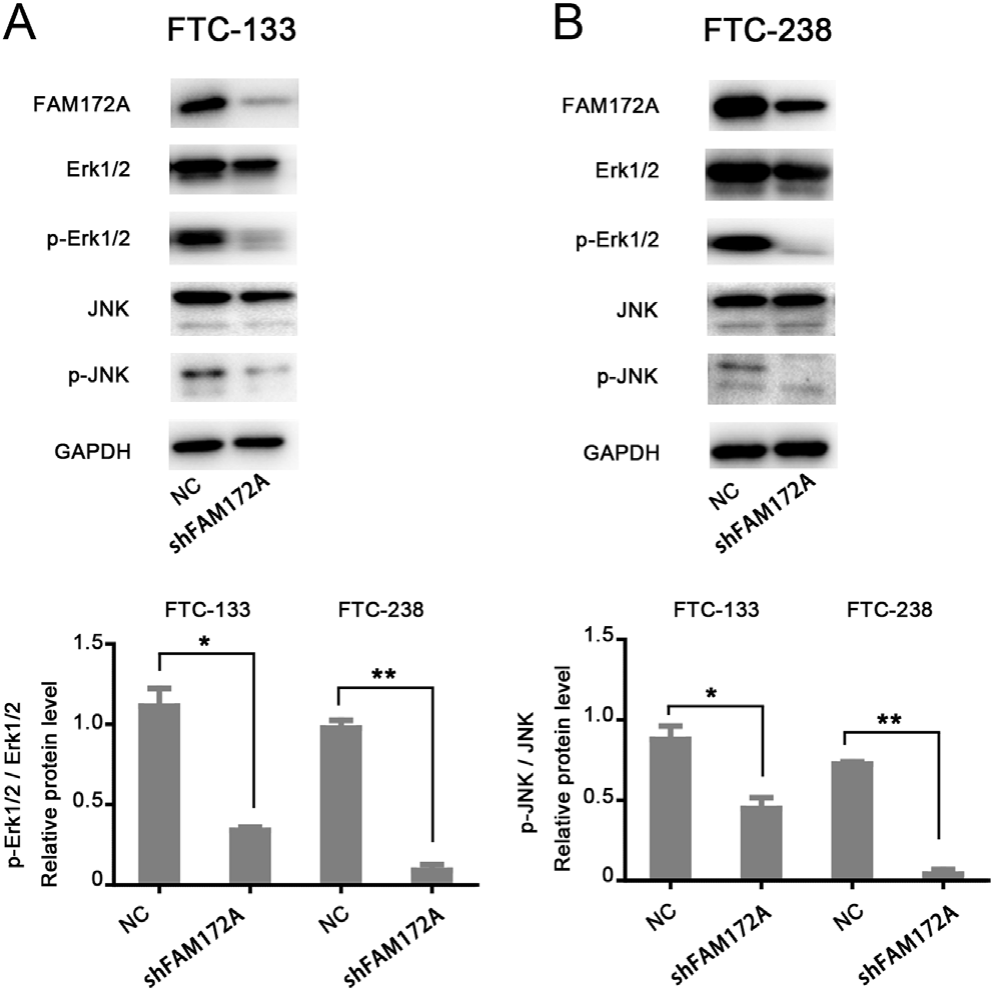
Western blot analysis involved in the influence of knockdown of FAM172A on MAPK signaling pathway. (A) The influence of FAM172A knockdown on the expression of Erk1/2, p- Erk1/2, JNK and p-JNK in the Erk1/2 and JNK MAPK pathway in FTC-133 cells. (B) The influence of FAM172A knockdown on the expression of Erk1/2, p- Erk1/2, JNK and p-JNK in the Erk1/2 and JNK MAPK pathway in FTC-238 cells. (Differences between two groups were analyzed using the independent t-test, values are expressed as the means ± s.e.m. *P < 0.05 and **P < 0.01**).

**Figure 6:**
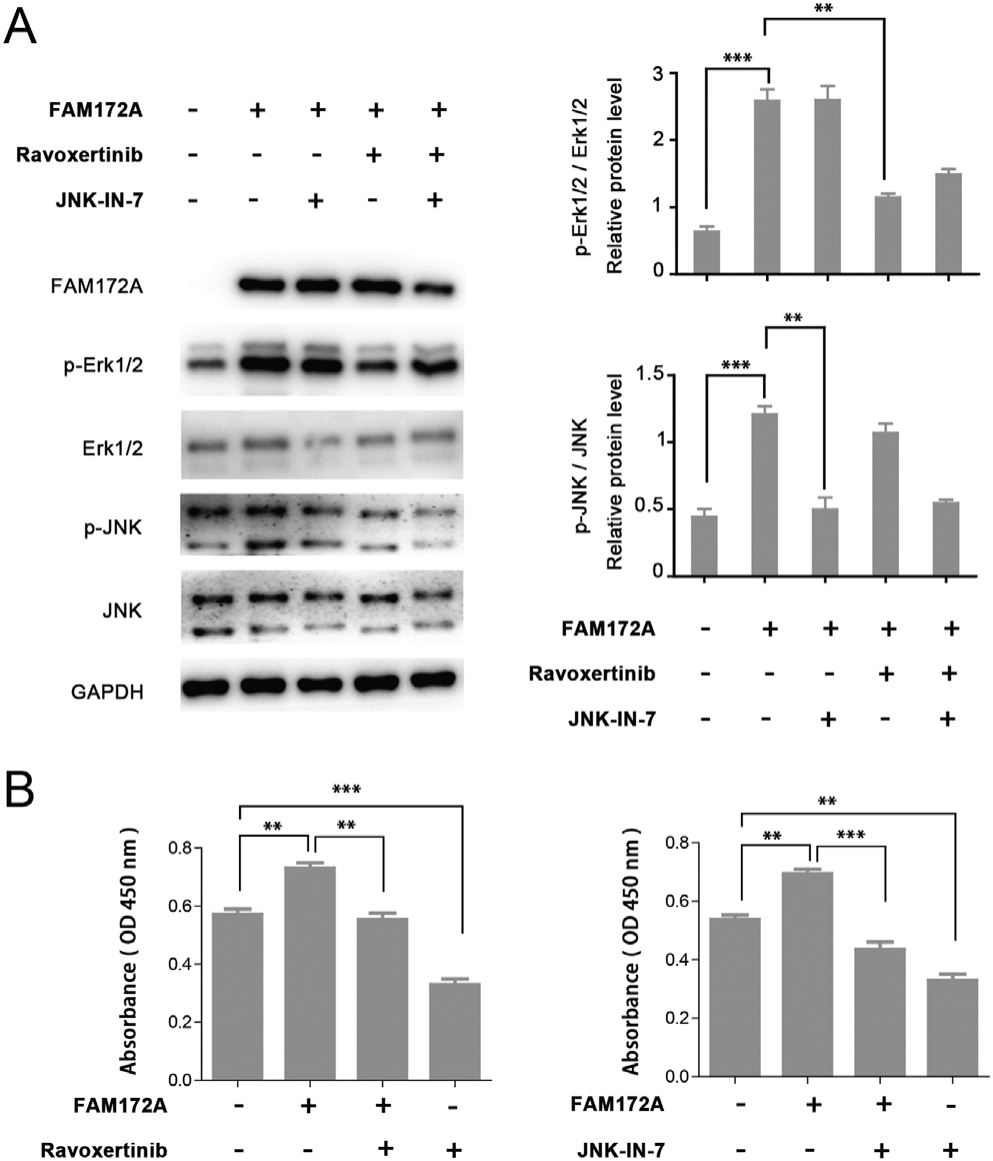
Western blot analysis of MAPK signaling pathway involved in the roles of FAM172A in FTC. (A) Western blot analysis of p-ERK and p-JNK expression in FTC-133 cells pretreated with overexpression of FAM172A, followed by treatment with ravoxertinib (Erk1/2 inhibitor) and JNK-IN-7 (JNK inhibitor) for 24 h. (B) The influence of ravoxertinib (Erk1/2 inhibitor) and JNK-IN-7 (JNK inhibitor) on the proliferation of FTC-133 cells with overexpression of FAM172A. (Differences between two groups were analyzed using the independent t-test, values are expressed as the means ± s.e.m. **P < 0.01 and ***P < 0.001).

To determine whether FAM172A plays a critical role through Erk1/2 and JNK MAPK pathways in the pathogenesis of FTC, we examined the changes of MAPK signaling pathway-related proteins including Erk1/2, p-Erk1/2, JNK and p-JNK using immunoblotting in both FTC-133 and FTC-238 cells. The results showed that downregulation of FAM172Asuppressed the expression of p-Erk1/2 and p-JNK in both FTC-133 and FTC-238 cells (Fig. 5A and B). Moreover, overexpression of FAM172A in FTC-133 significantly increased the expression of p-Erk1/2, which can be reversed by MAPK pathway specific-inhibitor ravoxertinib (Fig. 6A). Meanwhile, overexpression of FAM172A in FTC-133 significantly increased the expression of p-JNK, which can also be reversed by MAPK pathway specific-inhibitor JNK-IN-7 (Fig. 6A). Moreover, the effect of ravoxertinib and JNK-IN-7 on cell proliferation was investigated by CCK8 assay. The results showed that ravoxertinib and JNK-IN-7 significantly suppressed the proliferation of FTC-133 cells (*P* < 0.001 and *P* < 0.01, respectively) (Fig. 6B). Likewise, overexpression of FAM172A markedly promoted the proliferation of FTC-133 cells, while ravoxertinib and JNK-IN-7 could significantly reverse this effect (*P* < 0.01 and *P* < 0.001, respectively) (Fig. 6B).

Therefore, our findings indicated that FAM172A plays an important role in the pathogenesis of FTC through Erk1/2 and JNK MAPK pathways, which attributed to influencing the phosphorylation levels of Erk1/2 and JNK proteins.

### The value of FAM172A in the differential diagnosis of follicular thyroid lesions

To evaluate the potential value of FAM172A to differentiate malignant from other follicular thyroid lesions, retrospective IHC of FAM172A was performed with formalin-fixed paraffin-embedded (FFPE) slides from 120 patients with thyroid follicular tumors including 60 FTC, 30 FT-UMP and 30 FTA. The clinical and pathological characteristics are compared among the patients with FTC, FT-UMP and FTA, which are presented in Supplementary Table 1. The pathological features of FTC are also shown in Supplementary Table 1.

**Figure 7:**
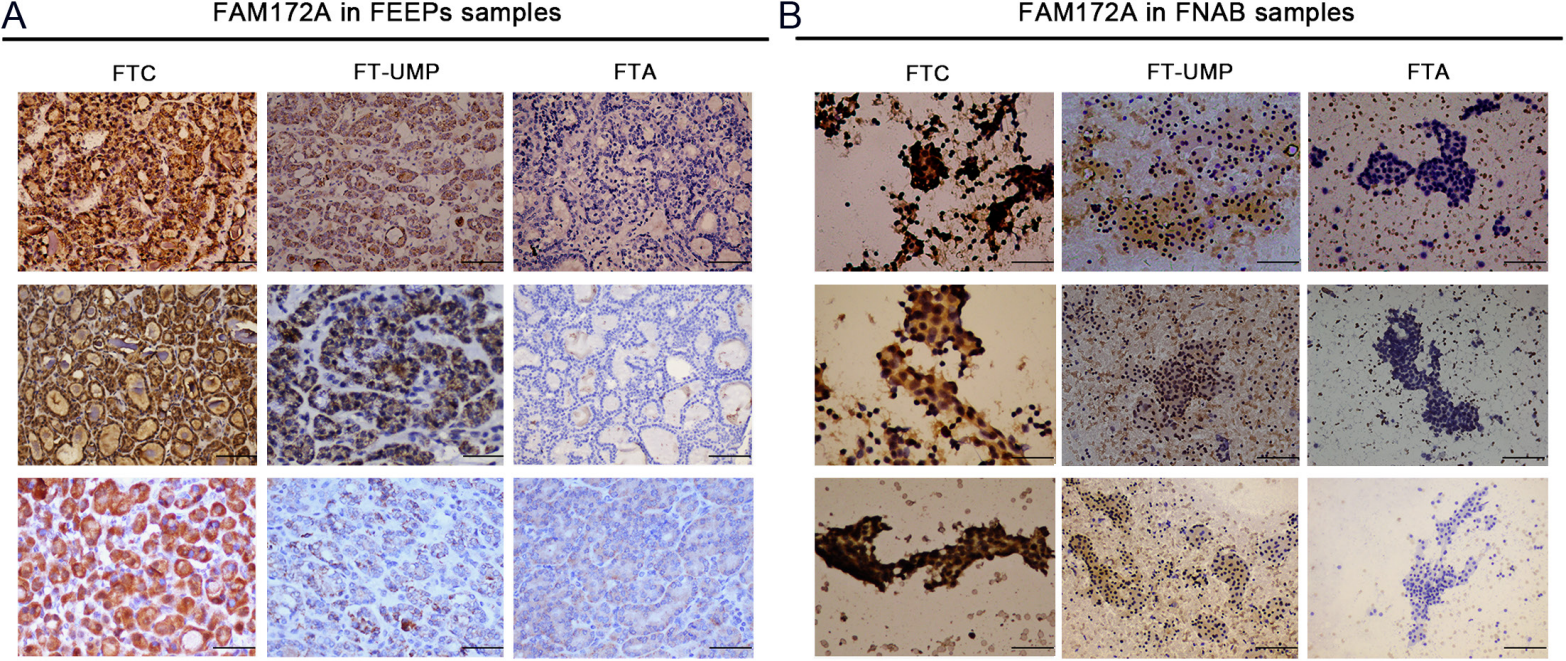
The comparison of expression of FAM172A among FTC, FT-UMP and FTA FFPEs and FNAB samples. (A) The comparison of IHC results of FAM172A among FTC, FT-UMP and FTA FFPEs samples. (B) The comparison of IHC results of FAM172A among FTC, FT-UMP and FTA FNAB samples.

Representative IHC staining images of FAM172A are shown in Fig. 7A and detailed results of IHC scores of FAM172A are presented in Supplementary Table 2. The IHC results found that the expression of FAM172A protein in FTC tissues was significantly higher than that in FT-UMP and FTA tissues (Fig. 7A). Likewise, the staining scores of FAM172A were significantly higher in the FTC than in the FT-UMP and FTA (Supplementary Table 2). To validate the specificity of FAM172A antibodies, IHC using two FAM172A antibodies from different companies was performed in normal human thyroid, FTA and FTC tissues. The results showed that the expression level of FAM172A is consistent using two different antibodies (Supplementary Fig. 4).

Based on the IHC staining scores of FAM172A, scores of 3.5 is the optimal cut-point with 92% sensitivity and 63% specificity to distinguish FTC from FT-UMP/FTA, and with 92% sensitivity and 80% specificity to distinguish FTC from FTA in FFPE specimens (Supplementary Fig. 5). According to this standard of 3.5 scores, 55 FTC cases were positive from 60 malignant FTC. Meanwhile, 14 FT-UMP cases were negative from 30 FT-UMP cases and 24 FTA cases were negative from 30 FTA cases (Table 1).

**Table 1:** The diagnosis by IHC scores of FAM172A in FFPEs and FNAB samples.

	IHC scores of FAM172A		Diagnostic value of FAM172A (excluding FT-UMP)			
	<3.5	≥3.5	Sensitivity (%)	Specificity (%)	PPV (%)	NPV (%)
FFPEs samples (120)						
FTC	5	55	92	80	90	83
FT-UMP	14	16				
FTA	24	6				
FNAB samples (81)						
FTC	3	9	75	89	64	93
FT-UMP	14	10				
FTA	40	5				

PPV, positive predictive value; NPV, negative predictive value.

To validate the value of FAM172A in the preoperative diagnosis of FTC, 81 patients with highly suspicious thyroid follicular tumors before the surgical operation were examined by FNAB. IHC of FAM172A was performed with all FNAB samples before the surgical operation. Based on postoperative pathological results, 81 FNAB samples were identified as 12 FTC, 24 FT-UMP and 45 FTA. The clinical characteristics of the patients and pathological features of the tissue samples are summarized in Supplementary Table 3. The pathological characteristics of FTC are also presented in Supplementary Table 3.

Examples of FAM172A IHC in FNA smears are presented in Fig. 7B. The IHC results showed that the expression of FAM172A was significantly higher in FTC compared with the other two groups of FANB samples. The IHC staining scores of FAM172A including score of positive staining percentage and score of staining intensity are presented in Supplementary Table 4. The results demonstrated that the scores of FAM172A were significantly higher in FTC (6.92 ± 3.96) than in FT-UMP (3.54 ± 2.59) and FTA (1.83 ± 2.21) (*P* < 0.001).

The preoperative diagnosis based on IHC scores of FAM172A was also presented in Table 1. IHC scores of FAM172A equal to or greater than 3.5 were considered as the presence of FTC, which effectively distinguished FTC from benign/ borderline thyroid tumors with 75% sensitivity and 78% specificity, respectively. If excluding borderline thyroid tumors, FAM172A will have 89% specificity to separate FTC from benign thyroid tumors. Additionally, FAM172A had 90% positive predictive value (PPV) and 83% negative predictive value (NPV) for the diagnosis of FTC. The final diagnosis of each case was determined by postoperative pathology. In detail, according to preset standards, 9 FTC cases were positive from 12 malignant FTC based on postoperative pathology. Meanwhile, 14 FT-UMP cases were negative from 24 FT-UMP cases and 40 FTA cases were negative from 45 FTA cases.

## Discussion

Currently, the diagnosis of FTC is generally based on the presence of capsule or/and vascular invasion (Netea-Maier *et al.* 2008). The preoperative diagnosis of FTC is difficult because of the lack of distinctive cytological features. Therefore, FTC cannot be distinguished from FTA based on cytological material obtained from FNAB, because capsular and vascular invasion are not available in cytology specimens (Grani *et al.* 2018). Base on The Bethesda System for Reporting Thyroid Cytopathology, thyroid follicular lesions were divided into two different categories: class 3 (atypia of undetermined significance/follicular lesion of undetermined significance (AUS/FLUS)) and class 4 (follicular neoplasm/suspicious for a follicular neoplasm (FN/SFN)), which only predict malignancy risk of thyroid follicular lesion approximately 5–15% (class 3) and 15–30% (class 4), respectively (Cibas & Ali 2009). Thus, in recent years, molecular diagnostics has become a promising approach to distinguish malignant from benign thyroid follicular neoplasms. As recommended by the latest guidelines from the ATA, molecular testing can be used to improve the assessment of malignancy risk, especially in thyroid follicular lesions (Haugen 2017).

Therefore, protein biomarker candidates for the diagnosis of FTC have been widely reported. Of these protein markers, some are involved in cell adhesion such as E-cadherin, and some associated with cell differentiation and malignant transformation like galectin-3, which to a certain extent contribute to the identification of thyroid epithelial malignancies (Herrmann *et al.* 2002, Kato *et al.* 2002, Mehrotra *et al.* 2004, Carpi *et al.* 2006, Bartolazzi *et al.* 2008). For example, galectin-3 has been extensively studied in thyroid follicular lesions (Herrmann *et al.* 2002, Mehrotra *et al.* 2004, Carpi *et al.* 2006, Bartolazzi *et al.* 2008). A study by Bartolazzi et al.** demonstrated that the overall sensitivity and specificity of the galectin-3 test was 78 and 93% in distinguishing malignant and benign follicular thyroid nodules, respectively (Bartolazzi *et al.* 2008). However, another study found that the galectin-3 was expressed not only in the majority of PTC and FTC, but also expressed in FTA, multinodular goiters, normal thyroid tissue and Hashimoto’s thyroiditis, which indicates that galectin-3 is not a reliable marker to differentiate malignant from benign thyroid follicular lesions (Mehrotra *et al.* 2004).

Therefore, new markers that contribute to the discrimination between malignant and benign thyroid follicular tumors are useful and necessary in clinical practice. FAM172A protein was confirmed for the first time by our team (Li *et al.* 2010). Furthermore, a recent study of ours found that FAM172A protein is significantly highly expressed in human PTC tissues and markedly promotes the proliferation of human PTC cell line IHH4 cells, which indicates that FAM172A may relate to the pathogenesis of PTC (Li *et al.* 2016). According to the Human Protein Atlas, FAM172A is widely expressed in all tissues, and moderately expressed in normal thyroid tissues, which indicates FAM172A may be an important protein related to normal growth of thyroid follicular epithelial cells. Meanwhile, FAM172A is highly expressed in thyroid tumor cells and tissues, and it is speculated that the activation of FAM172A may play vital roles in cell proliferation and invasion and tumor growth.

A study by Bélanger *et al.* demonstrated that Fam172a plays a key role in the pathogenesis of CHARGE syndrome (BéLanger *et al.* 2018). Furthermore, sequencing data in this study showed that Fn1 (Fibronectin1) may be an important gene related to Fam172a. Interestingly, FN1 is highly expressed in FTC and closely related to the pathogenesis of FTC (Prasad *et al.* 2005), which indicated the important role of FAM172A in the pathogenesis of FTC.

Therefore, based on our previous studies, we speculated whether FAM172A also plays an important role in the carcinogenesis of FTC.

Consistent with our previous results (Li *et al.* 2010), FAM172A was also mainly located in the nucleus of FTC cell (Supplementary Fig. 1C). Intriguingly, the expression of FAM172A was also significantly higher in human FTC tissues than in peri-carcinoma, FT-UMP and FTA tissues in the present study. Furthermore, FAM172A expression in human FTC cells was also clearly higher than that in normal human thyroid cells. Therefore, based on the significantly high expression of FAM172A in FTC tissues and cells, we speculated whether FAM172A may be considered as a useful diagnostic marker for FTC before the surgical operation, which was investigated and assessed using postoperative FFPE and preoperative FNA thyroid samples.

First, the quantitative evaluation of IHC scores of FAM172A staining were performed from 120 FFPE samples based on the positive staining percentage and staining intensity. The optimal cut-off point of FAM172A IHC scores to discriminate FTC from benign/borderline thyroid follicular tumors was 3.5 with 92% sensitivity and 63% specificity using ROC curve analysis. Presently, the sensitivity and specificity of protein markers distinguishing malignant from benign thyroid follicular tumors range from 60 to 86%, and from 36 to 89%, respectively (Mehrotra *et al.* 2004, Jang *et al.* 2015, Lai *et al.* 2017). Compared with previous studies, the sensitivity of identifying FTC from thyroid follicular tumors was similar between FAM172A and other markers. The specificity is slightly lower in our study than in other studies, which may attribute to the recruitment of the borderline thyroid tumor (FT-UMP) into the present study. However, excluding FT-UMP, IHC scores of 3.5 of FAM172A will have 92% sensitivity and 80% specificity to separate FTC from FTA. Therefore, unlike many previous studies that only included FTC and FTA, FT-UMP was also observed in our present study given that FT-UMP is frequently encountered in clinical practice.

FT-UMP is one of the borderline/precursor thyroid tumors between malignant and benign thyroid follicular lesions, which is characterized by questionable/incomplete capsular invasion but without vascular invasion or nuclear changes (Kakudo 2018). A previous study by Piana et al.** observed no occurrence of cancer death in 6 patients with FT-UMP on average 11.9 years’ follow-up (Piana *et al.* 2010), which indicates that FT-UMP may be treated with careful follow-up without surgery. Thus, FTA and FT-UMP were classified as the same category, and FTC another category in the present study. Our results showed that the expression of FAM172A in FTC tissues not only significantly higher than that in FTA tissues, but also obviously higher than that in FT-UMP tissues, which indicated that as a marker, FAM172A may distinguish FTC from FTA and FT-UMP. However, our previous study found that FAM172A was also highly expressed in PTC tissues (Li *et al.* 2016). Therefore, it may be difficult to discriminate FTC from PTC only using IHC analysis. However, FAM172A test combined with ultrasound examination of thyroid and fine-needle aspiration biopsy will be helpful to distinguish FTC from PTC.

Therefore, the current high expression of FAM172A can distinguish PTC and FTC from other benign tumors. Furthermore, the identification of PTC and FTC requires the combination of ultrasound and fine-needle aspiration biopsy.

In order to further verify the value and effectiveness of FAM172A in identifying FTC from thyroid follicular lesions, 81 FNA samples were used to perform the preoperative diagnosis of thyroid follicular lesions according to IHC scores of FAM172A. Similar to the results from FFPE samples, IHC scores of 3.5 of FAM172A still retain sufficient and high sensitivity (75%) and specificity (78%) to discriminate malignant from benign/borderline thyroid tumors using FNA samples. Interestingly, the specificity increased to 89% after excluding FT-UMP samples. A previous prospective study found that HBME-1 is an excellent marker for discriminating malignant from benign thyroid tumors with 79% sensitivity and 84% specificity using 418 preoperative thyroid FNA samples (Franco *et al.* 2009). Compared with HBME-1, FAM172A had similar sensitivity and specificity to distinguish FTC from benign/borderline thyroid tumors using preoperative thyroid FNA samples.

Subsequently, the role and mechanism of FAM172A in the pathogenesis of FTC were primarily studied. The over-proliferation, migration and invasion of cell are important features of malignant tumors, and play a crucial role in the development and progression of cancers. Consistently, our study found that the knockdown of FAM172A gene significantly inhibited the proliferation, migration and invasion of human FTC cells. Contrarily, the overexpression of FAM172A gene obviously promoted the proliferation and migration of normal thyrocytes. Furthermore, *in vivo* experiments in nude mice also showed that the knockdown of FAM172A gene markedly suppressed the tumorigenicity of human FTC cells. These findings indicated that FAM172A was closely associated with the pathogenesis and carcinogenesis of FTC and may be a novel tumor promoter gene of FTC. Finally, based on the findings of next-generation sequencing, mitogen-activated protein kinase (MAPK) signaling pathway was chosen to investigate the possible mechanisms of FAM17A in the pathogenesis of FTC. Mutation or rearrangements of genes associated with MAPK pathway effectors maybe required for the malignant transformation of thyroid follicular cells (Kondo *et al.* 2006). For example, mutation of BRAF gene was one of the most common MAPK effectors in thyroid tumorigenesis (Qu *et al.* 2017). Furthermore, the oncogenic activation of MAPK signaling in thyroid follicular cells further increases genomic instability and leads to somatic mutations during FTC progression. Therefore, MAPK signaling pathway is closely linked with the occurrence and development of thyroid malignant tumors (Saavedra *et al.* 2000, Ciampi *et al.* 2005). Thus, MAPK pathway components including Erk1/2 and JNK were studied to clarify the role of FAM172A in the pathogenesis of FTC.

The present results showed that downregulation of FAM172A suppressed the expression of p-Erk1/2 and p-JNK in FTC cells. Contrarily, the overexpression of FAM172A increased the expression of p-Erk1/2 and p-JNK. Furthermore, ravoxertinib (Erk1/2 inhibitor) and JNK-IN-7 (JNK inhibitor) can reverse the expression of p-Erk1/2 and p-JNK in cell lines with FAM172A overexpression. All results indicated that FAM172A may regulate Erk1/2 and JNK pathways by influencing the protein phosphorylation levels. It is generally believed that both Erk1/2 and JNK pathways play important roles in cell proliferation and tumorigenesis. Therefore, the activation of Erk1/2 and JNK pathways may be one of the mechanisms of FAM172A in the pathogenesis and carcinogenesis of FTC.

However, the value and reliability of FAM172A in preoperative diagnosing FTC need further extensive evaluation and confirmation with more cases from multicenter. In addition, whether other thyroid diseases such as Hashimoto’s thyroiditis may influence the expression of FAM172A in thyroid also need further assessment in the future.

## Conclusions

In summary, FAM172A is highly expressed in FTC tissues, and plays an important role in the carcinogenesis of FTC via Erk1/2 and JNK pathways. Based on FANB and IHC, FAM172A may be a potential molecular marker to differentially diagnose FTC and benign/borderline thyroid follicular lesions. The precise role and mechanism of FAM172A in the pathogenesis of FTC should be further studied in the future. Additionally, future prospective clinical investigations should be considered and conducted to validate the values of FAM172A in preoperative diagnosing FTC in a larger cohort of FNAB samples.

## Supplementary Material

Materials and methods

Supplementary Figure 1. The isoforms and localization of FAM172A and the relationship between the expression level of FAM172A and Ki-67 (A) The expression of FAM172A isoforms (isoform1 and isoform2) detected by western blot in thyroid cells. (B) The relationship between the expression level of FAM172A and Ki-67. (C) The cell localization of FAM172A protein detected by immunofluorescence staining in Nthy-ori 3-1 and FTC-133 cells.

Supplementary Figure 2. Potentially important genes associated with FAM172A in FTC. (A) The heat-map profile of differentially expressed genes in Nthy-ori 3-1 cells with and without overexpression of FAM172A. Green-spots represent down-regulated genes and red-spots represent up-regulated genes. (B) The heat-map profile of differentially expressed genes between FTC and peri-carcinoma tissue samples. (C) Overlapping 61 differentially expressed genes between two comparisons were demonstrated with Venn diagrams. (D) Five potentially important genes associated with FAM172A were verified in FTC cell lines with knockdown and overexpression of FAM172A.

supplementary Figure 3. MAPK Signaling pathway associated with FAM172A in FTC. KEGG pathway for MAPK signaling. Differential expression of genes was marked red (p values <0.05).

Supplementary Figure 4. Comparison of expression of FAM172A detected by IHC in FFPE samples using two different FAM172A antibodies.

supplementary Figure 5. ROC curve of FFPE samples. (A) ROC curve of FFPE samples (n=120) with cut-point based on IHC scores of FAM172A discriminating FTC and benign/borderline thyroid follicular lesions. (B) ROC curve of FFPE samples (n=90) with cut-point based on IHC scores of FAM172A discriminating FTC and benign thyroid follicular lesions.

Table S1 Clinical and pathological characteristics in 120 FFPEs samples

Table S2 The IHC staining scores of FAM172A from FFPEs samples

Table S3 Clinical and pathological characteristics in 81 FNAB samples

Table S2 The IHC staining scores of FAM172A from FNAB samples

## Declaration of interest

The authors declare that there is no conflict of interest that could be perceived as prejudicing the impartiality of the research reported.

## Funding

This study was supported by grants from the National Key Research and Development Plan (2018YFC1314900 and 2018YFC1314905), the National Natural Science Foundation of China (81170759, 81502316, 81672373, 81770813 and 82070866), and the Science and Technology Commission of Shanghai Municipality (15411960600 and 14411964100).
