# Supplementary material for: FAM172A promotes follicular thyroid carcinogenesis and may be a marker of FTC
Source: Endocr Relat Cancer. 2020 Sep 21;27(11):657–69. doi: 10.1530/ERC-20-0181 (PMC7707803; doi:10.1530/ERC-20-0181)
Supplement: Materials and methods [file supplementary_material.pdf]

## Materials and methods

### Case selection

Human FTC tumor tissues and adjacent non-tumor tissues were collected from six patients with confirmed FTC. The tissue samples were snap-frozen in liquid nitrogen immediately after resection and subsequently were stored at -80°C. 120 FFPE samples were obtained from pathology department of Shanghai Sixth People's Hospital during the period from January 2013 to January 2017. 81 patients underwent FNAB were included from surgery department of Shanghai Sixth People's Hospital from January 2017 to August 2018.

The clinical and pathological data of the studied cases were collected using the medical record system. The study was approved by the ethics committee of Shanghai Jiao Tong University Affiliated Sixth People's Hospital, and written informed consent was obtained from all patients.

### IHC analysis

The expression levels of FAM172A were assessed by IHC analysis. The slides were heated at 60°C for 40 minutes and dewaxed and rehydrated for antigen retrieval with 10nM citrate antigen repair solution (Sangon Biotech, Shanghai, China) 10 minutes at 95°C. FAM172A rabbit polyclonal antibody (Abcam, Cambridge, USA) was used at a dilution of 1:400 in FFPE samples and 1:200 in FNAB samples. Ki-67 rabbit polyclonal antibody (Abcam, Cambridge, USA)) was used at a dilution of 1:200. The slides were incubated with primary antibodies overnight at 4°C, followed by incubation with secondary biotinylated antibody for 30 minutes at 37°C.

Based on the percentage and intensity of the positively stained tumor cells under microscopy (400X), a semi-quantitative scoring system was applied to assess the staining results (Douwes Dekker et al., 2007, Bovee et al., 2006, Pansuriya et al., 2011, Hinkel et al., 2008, Lai et al., 2017). Finally, the percentage score × the intensity score ≥ 3.5 was considered as a standard to prospectively predict the presence of FTC in FNAB samples. IHC evaluation of FAM172A was performed by a proficient pathologist using uniform and pre-established criteria.

To validate the specificity of FAM172A rabbit polyclonal antibody (Abcam, Cambridge, USA), another FAM172A antibody (Atlas antibodies, AB, Stockholm, Sweden) was used in IHC at a dilution of 1:400 in FFPE sample.

**Cell culture**

Nthy-ori 3-1 cells were derived from normal thyroid tissue cells transfected with a plasmid encoding the SV40 large T gene. Human thyroid follicular carcinoma cell lines FTC133 and FTC 238 were originated from the same patient with FTC. FTC133 was derived from a primary thyroid tumor and FTC 238 from a lung metastasis. Nthy-ori 3-1 (passage number 12) and FTC-133 (passage number 15) cell line was gifted from Dr. Yuchen Jin and Dr. Weijun Wei (Shanghai Jiao Tong University). FTC-238 (passage number 10) cell line was purchased from the ECACC Company (Shanghai, China). All cell lines including Nthy-ori 3-1, FTC-133 and FTC-238 were maintained in RPMI 1640 medium (Gibco, Grand Island, NY, USA) supplemented with 10% fetal bovine serum (FBS, Gibco, Grand Island, NY, USA). Cells were maintained at 37°C in a humidified 5% CO2 incubator. All cell lines were

authenticated using Short Tandem Repeat (STR) analysis.

**RNA extraction and RT-PCR**

RNA extraction from cells was performed by TRIZOL reagent according to a standard protocol. A total of 1 μg DNase-treated RNA was reverse-transcribed into cDNA using the QuantiTect Reverse Transcription Kit (QIAGEN, Valencia, CA, USA). Two negative controls were set in the experiment including one without template RNA and the other without reverse transcriptase. Meanwhile, overexpression of FAM172A plasmid was used as a positive control. The mRNA levels of FAM172A were quantified by SYBR-Green q-PCR (Takara, Otsu, Shiga, Japan) and normalized to the levels of GAPDH. The potentially important genes associated with FTC including DCN, ABCC3, PCSK6, FXYD6, IGFBP3 were also quantified by SYBR-Green q-PCR and normalized to the levels of GAPDH. The sequences of upstream and downstream primers were as follows: FAM172A, 5′-TGA ACC GCC TCT TGA TTT TCC-3′ and 5′- AGA GCC TCG TAT CTT TTC TGG T-3′; DCN, 5′-ATG AAG GCC ACT ATC ATC CTC C-3′ and 5′-GTC GCG GTC ATC AGG AAC TT-3′; ABCC3, 5′-TGG GGT GAA GTT TCG TAC TGG-3′ and 5′- CAC GTT TGA CTG AGT TGG TGA TA-3′; PCSK6, 5′-CGC AGG CCC TTT ACT TCA AC-3′ and 5′- CGG CAG CGA CTG TTC TTG T-3′; IGFBP3, 5′-AGA GCA CAG ATA CCC AGA ACT-3′ and 5′- GGT GAT TCA GTG TGT CTT CCA TT-3′; GAPDH, 5′-TGT TGC CAT CAA TGA CCC CTT-3′ and 5′- CTC CAC GAC GTA CTC AGC G-3′. All PCR reactions were performed in triplicate. All independent experiments were performed in triplicate.

**Immunoblotting**

Total proteins were electrophoresed in 10% polyacrylamide gels and transferred to NC membranes (Millipore, Bedford, UK). The electrophoresis was run in running buffer at 90V for 0.5h and then 120V for 1.5h. Blots were blocked for 1h in 5% milk/0.05% tris-buffered saline tween (TBS-T) and then incubated with primary antibodies at 4°C overnight. The blots were then washed four times in TBS-T for 20 minutes and then incubated with secondary antibodies (HRP-conjugated goat anti-mouse, goat anti-rabbit and rabbit anti-goat IgG; 1:5000, Santa Cruz, Dallas, TX USA) in 5% milk/TBS-T for 1 hour, then washed 4 times in TBS-T for 5 minutes each time. The membrane was visualized with ECL detection reagent (Amersham Biosciences, Castle Hill, Australia) and then exposed on X-ray film. Antibodies against FAM172A (1:1000, Abcam, USA), Erk1/2, p-Erk1/2, JNK, p-JNK, and GAPDH (1:1000, Cell Signaling Technology, USA) were applied for immunoblotting. All data were performed in triplicate. All independent experiments were performed in triplicate.

**Establishment of stable cell lines of FAM172A overexpression and downregulation**

Human FAM172A gene sequence was cloned into lentiviral vector PDS159_pL6.3-CMV-GFPa1-IRES-MCS to generate the PDS159-FAM172A plasmid. The lentivirus packaging system consists of 3 plasmids: PDS159-FAM172A, pCMV-dR8.91 (Delta 8.9) and pCMV-VSV-G. The three plasmids were co-transfected into 293T cells with a 4:2:1 ratio to package lentiviral particles. After

36h and 60h transfection, the supernatants were harvested, concentrated and stored at -80°C. Infected cells were maintained at 37°C/5% CO2 for 48 h. Polyclonal cell populations were then cultured with blasticidin (5μg /ml for Nthy-ori 3-1 and FTC-133) to allow for selection of blasticidin-resistant cells. Human FAM172A specific shRNA (TRC shRNA TRCN0000127701) and control shRNA (TRC shRNA TRCN0000072223) were constructed according to pLKO.1 Protocol (Root et al., 2006). The method for packaging of the lentiviral particles was the same as above. Polyclonal cell populations were then cultured in puromycin (0.5μg/ml for FTC-133 and 1μg/ml for FTC-238) to allow for selection of puromycin-resistant cells. The TRC clone IDs and target sequences were as follows: shLacZ/ TRCN0000072223, TGT TCG CAT TAT CCG AAC CAT; and shFAM172A/ TRCN0000127701, GGA TAA ACA TGG CAC AAA TCC.

**Cell proliferation and colony formation assays**

Cell Counting Kit-8 (CCK8) (Biotool, Houston, TX, USA) was used as the end point to quantitatively assess the proliferation ability of Nthy-ori 3-1, FTC-133 and FTC-238 cells. Cells were seeded in 96-well plates at 2 x $10^3$ cells per well in triplicate for each condition. The CCK8 solution was added (10ul) to each well and incubated at 37°C/5% CO2 for 1 h. OD value was measured. The absorbance of each well was quantified at 450 nm on a microplate reader (168–1000 Model 680, Bio-Rad, Hercules, CA, USA).

The soft agar colony formation assay was applied to evaluate the ability of a single cell to grow into a colony. Cells were seeded into 6-well plates at 300 cells per plate.

The cells were mixed and then cultured for 7-10 days in culture media with 10% FBS. Cell clusters of more than 30 cells were counted as a colony. All independent experiments were performed in triplicate.

**Transwell assay**

Transwell assay was performed with 8μm polycarbonate transwell filters (Corning, Cambridge, USA). A total of 5 x $10^5$ cells were serum-starved overnight, and then were inoculated into each insert well in serum-free media (without Matrigel in migration assay, and with Matrigel in invasion assay), while 600μL media supplemented with 10% FBS were placed the well below. Matrigel (Corning, Cambridge, USA, 356234) was diluted 1:4. After 24h incubation, cells on the lower membrane were fixed in 4% paraformaldehyde and stained with crystal violet. Then, cells that had migrated onto the lower surface of the porous membrane were photographed and counted with an inverted microscope**.**

**Tumorigenicity assay in vivo**

Male *BALB/c* nude mice aged 4 weeks were purchased from Shanghai Experimental Animal Center (SLAC, China) and housed in a dedicated SPF facility at the Experimental Animal Center of the Sixth People's Hospital affiliated to Shanghai Jiaotong University. After two weeks, a total of $5\times10^6$ FTC-133 and FTC-238 cells stably infected with shRNA-FAM172A lentivirus particles were injected into the right groin of nude mice, respectively. The length and width of the resulting tumors were measured every 3 days (FTC-238 cells) or 5 days (FTC-133 cells) after injection. Then, nude mice were sacrificed to evaluate the xenograft performance in 21 days

(FTC-238 cells) or 30 days (FTC-133 cells), respectively. The xenograft tumors were collected and weighed. Xenograft tumor volumes were calculated as (length x width$^2$)/2. Animal care and experiments were approved by the committee for humane treatment of animals at Shanghai Jiao Tong University Affiliated Sixth People's Hospital.

**Next-generation sequencing technology**

The next generation sequencing work was performed by Amplicon-gene Bioscience (Shanghai, China) (https://www.amplicongene.com). Total RNA was extracted with TRIZOL reagent (sigma) following the recommendations of the manufacturer. RNA integrity was examined with the Bioanalyzer 2100 (Agilent). One pair of vector-Nthy-ori 3-1 and FAM172A-Nthy-ori 3-1 cell samples and three pairs of FTC and peri-carcinoma tissue samples were sequenced. Each sample was sequenced to a depth of approximately 45 million reads. For sequencing, the RNA-seq libraries were prepared according to the standard Illumina protocol with the mRNA-seq Illumina TruSeq. cDNA libraries were checked for quality and quantified on a 2100 Bioanalyzer (Agilent). Each library was sequenced with the Illumina Sequencing Kit on one lane of a HiSeq sequencer to obtain 150-bp paired-end reads. Quantile normalization and subsequent data processing were performed using the R software package (R version 3.1.2).

**The signaling pathways associated with the role of FAM172A in FTC**

Various stable transfected cell lines with knockdown and overexpression of FAM172A were maintained in RPMI 1640 medium, supplemented with 10% fetal

bovine serum. FAM172A knockdown cell lines were maintained with puromycin (0.5μg/ml for FTC-133 and 1μg/ml for FTC-238), and FAM172A overexpression cell lines with blasticidin (5μg/ml for FTC-133) respectively. After either 24 or 48 h of culture, cells were harvested followed by detect the expression of p-JNK, JNK, p-Erk1/2 and Erk1/2 by immunoblotting. To further determine the mechanisms of FAM172A involved in MAPK pathways, JNK inhibitor (JNK-IN-7, 1μM, TargetMol, USA) and Erk1/2 inhibitor (Ravoxertinib, 1μM, TargetMol, USA) were also added into the culture medium. After 24 h of culture, cells were then collected to detect the expression of p-JNK, JNK, p-Erk1/2 and Erk1/2 by immunoblotting.

**Statistical Analysis**

Where applicable, data were presented as the mean±SEM from at least three replicates. All independent experiments were performed in triplicate. Clinical data were expressed as mean±S.D, percentages or medians. Differences between two groups were analyzed using the independent t-test. Continuous variables associated with tumor characteristics were compared among three groups using one-way ANOVA test. Differences were considered statistically significant when P values were less than 0.05. All data were analyzed using SPSS13.0 statistical software (SPSS Inc., Chicago, IL, USA).
