# Supplementary figures and images for: FAM172A promotes follicular thyroid carcinogenesis and may be a marker of FTC
Source: Endocr Relat Cancer. 2020 Sep 21;27(11):657–69. doi: 10.1530/ERC-20-0181 (PMC7707803; doi:10.1530/ERC-20-0181)

**A**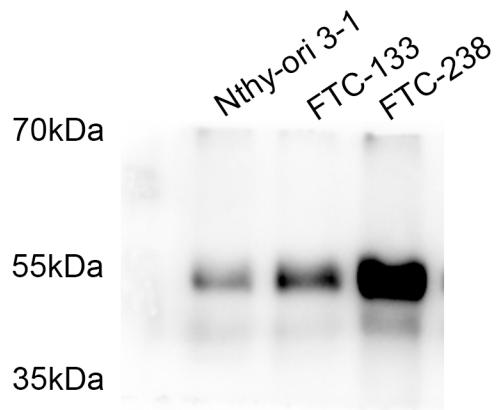**B**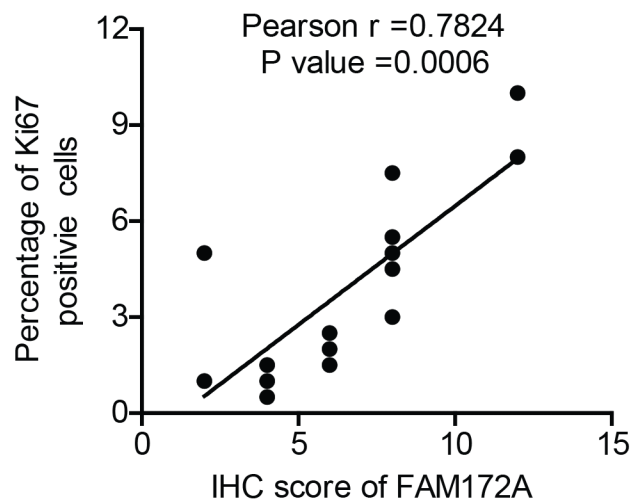**C**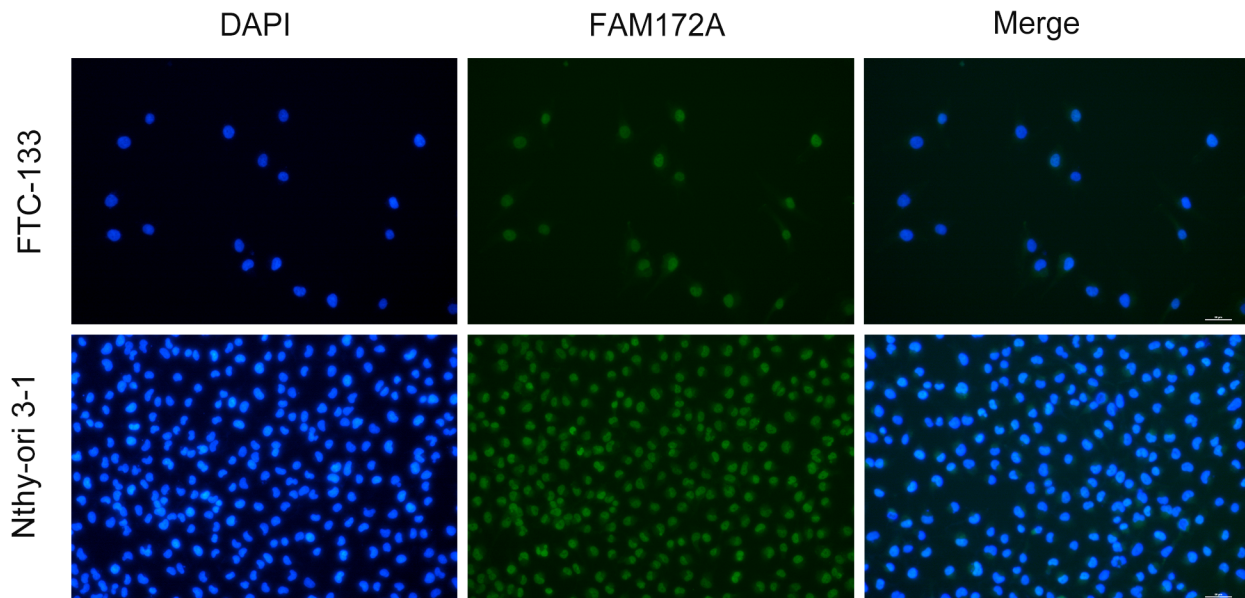

Supplement: Supplementary Figure 1. The isoforms and localization of FAM172A and the relationship between the expression level of FAM172A and Ki-67 (A) The expression of FAM172A isoforms (isoform1 and isoform2) detected by western blot in thyroid cells. (B) The relationship between the expression level of FAM17 [file supplementary_figure_1.pdf]

C

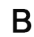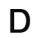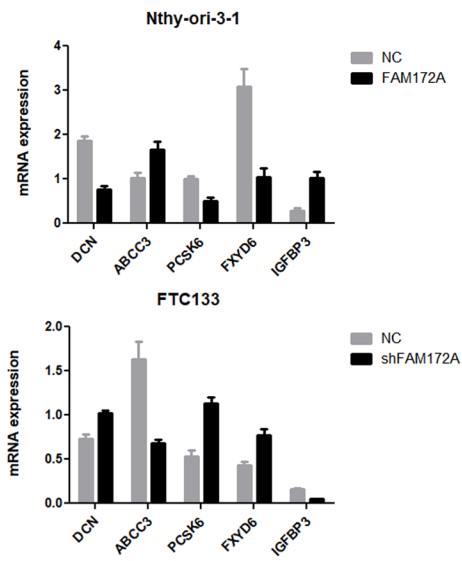

Supplement: Supplementary Figure 2. Potentially important genes associated with FAM172A in FTC. (A) The heat-map profile of differentially expressed genes in Nthy-ori 3-1 cells with and without overexpression of FAM172A. Green-spots represent down-regulated genes and red-spots represent up-regulated genes. (B)  [file supplementary_figure_2.pdf]

# MAPK SIGNALING PATHWAY

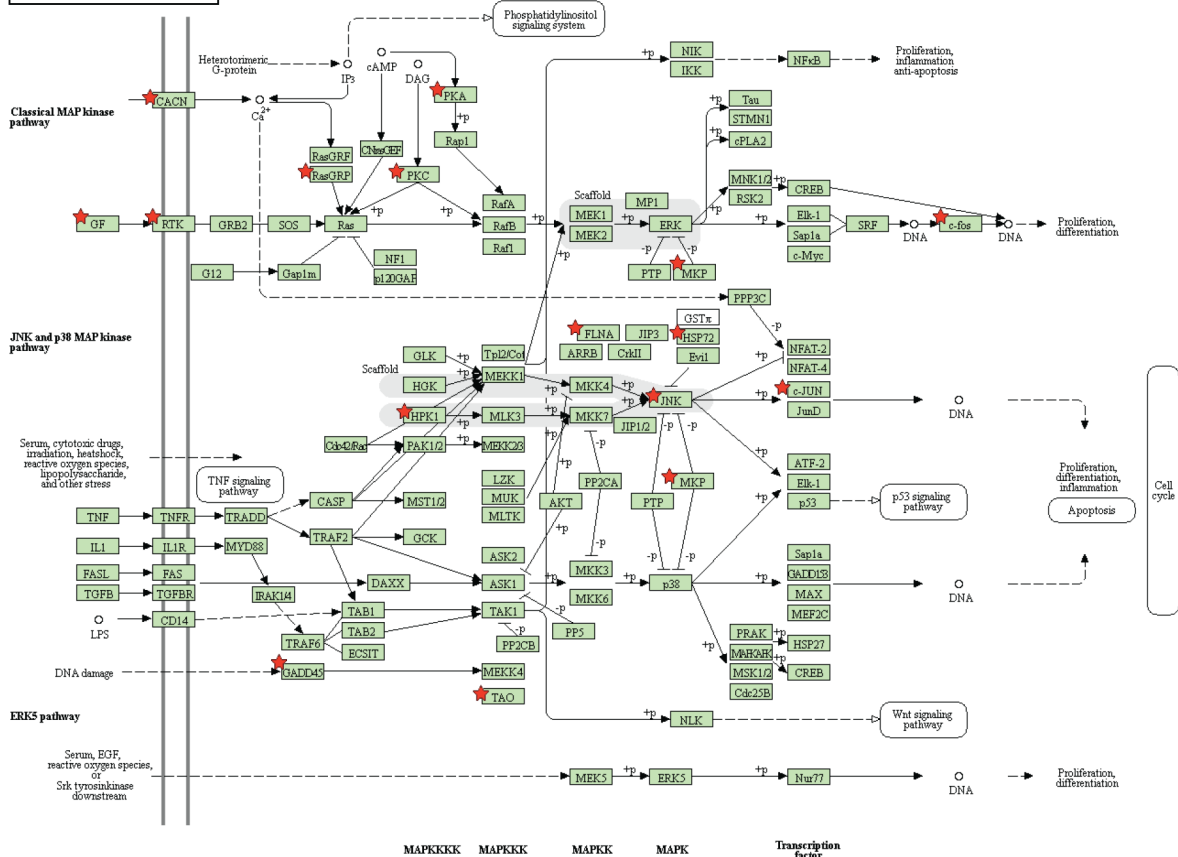

Supplement: supplementary Figure 3. MAPK Signaling pathway associated with FAM172A in FTC. KEGG pathway for MAPK signaling. Differential expression of genes was marked red (p values <0.05). [file supplementary_figure_3.pdf]

**A**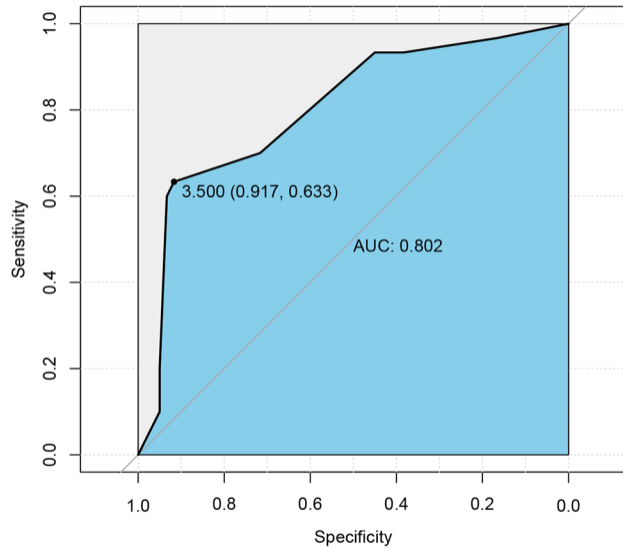**B**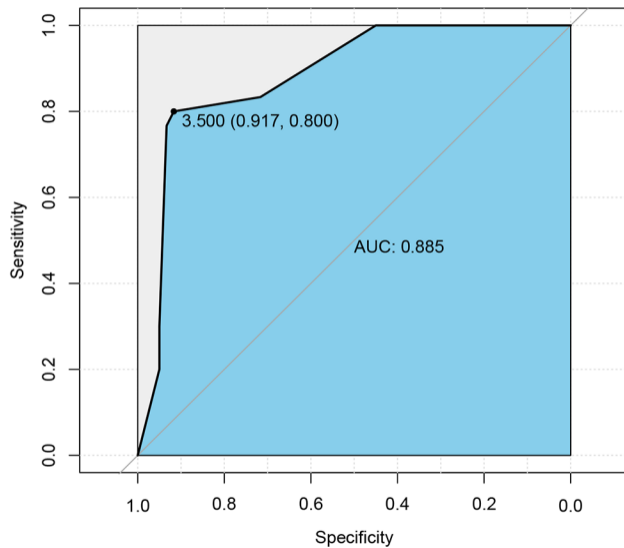

Supplement: supplementary Figure 5. ROC curve of FFPE samples. (A) ROC curve of FFPE samples (n=120) with cut-point based on IHC scores of FAM172A discriminating FTC and benign/borderline thyroid follicular lesions. (B) ROC curve of FFPE samples (n=90) with cut-point based on IHC scores of FAM172A discriminatin [file supplementary_figure_5.pdf]
