# Supplementary material for: FAM172A promotes follicular thyroid carcinogenesis and may be a marker of FTC
Source: Endocr Relat Cancer. 2020 Sep 21;27(11):657–69. doi: 10.1530/ERC-20-0181 (PMC7707803; doi:10.1530/ERC-20-0181)
Supplement: Supplementary Figure 4. Comparison of expression of FAM172A detected by IHC in FFPE samples using two different FAM172A antibodies. [file supplementary_figure_4.pdf]

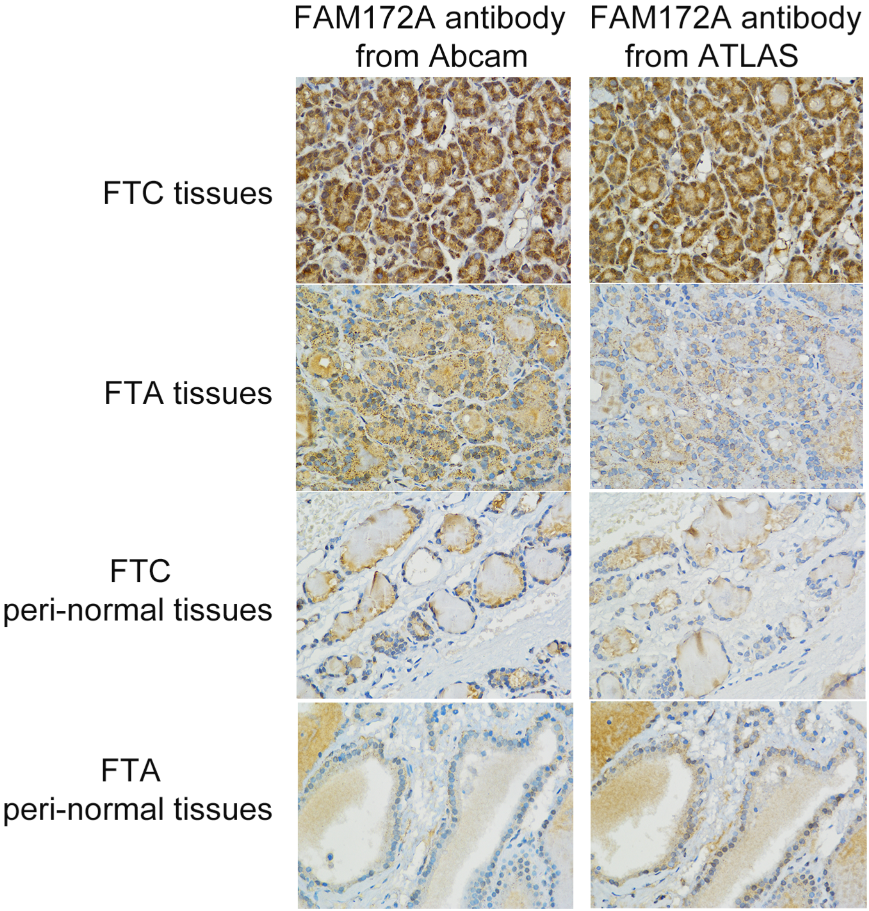
FAM172A antibody from Abcam
FAM172A antibody from ATLAS
FTC tissues
FTA tissues
FTC peri-normal tissues
FTA peri-normal tissues
