# Supplementary material for: FAM172A promotes follicular thyroid carcinogenesis and may be a marker of FTC
Source: Endocr Relat Cancer. 2020 Sep 21;27(11):657–69. doi: 10.1530/ERC-20-0181 (PMC7707803; doi:10.1530/ERC-20-0181)
Supplement: Table S1 Clinical and pathological characteristics in 120 FFPEs samples [file supplementary_table_1.pdf]

Table S1 Clinical and pathological characteristics in 120 FFPEs samples

Clinicopathological variables	FTC (60)	FT-UMP (30)	FTA (30)	P value
Age (years)	47±16	48±13	54±12	0.099
<55 (n, %)	37(61.67%)	18(60.00%)	14(46.67%)	0.204
Male (n, %)	19(31.67%)	5(16.67%)	3(10.00%)	0.015
*Time of diagnosis (month)	6(1-21)	5(2-18)	3(1-12)	0.347
*FT3 (pmol/L)	4.82(4.36-5.46)	4.86(4.33-5.51)	4.54(4.12-5.30)	0.598
*FT4 (pmol/L)	15.63(12.74-17.47)	15.48(14.89-16.77)	16.72(14.07-18.64)	0.875
*TSH (mIU/l)	2.09(1.19-3.11)	2.38(1.20-3.35)	1.70(1.13-2.54)	0.422
*TG (ng/ml)	94.22(55.23-461.26)	28.95(15.54-1226.48)	38.43(19.53-70.18)	0.155
*TGAb (KIU/L)	15.27(13.54-17.67)	15.03(11.41-163.02)	13.6(9.56-16.32)	0.138
*TPOAb (KIU/L)	5.29(5.00-7.71)	14.10(11.42-34.36)	8.56(7.65-9.25)	0.628
Single nodule (n, %)	13(21.67%)	12(40.00%)	5(16.67%)	0.900
*Tumor maximum diameter (cm)	3.0(2.0-4.0)	3.0(2.0-3.2)	1.9(1.1-3.2)	0.043
Minimally invasive (n, %)	32(53.33%)	-	-	-
Angioinvasion (n, %)	21(35.00%)	-	-	-
Lymph node metastasis (n, %)	6(10.00%)	-	-	-
Distant metastases (n, %)	4(6.67%)	-	-	-
Extrathyroidal invasion (n, %)	1(1.67%)	-	-	-
Combined with PTMC (n, %)	2(3.34%)	2(6.67%)	7(23.33%)	0.003
Combined with HT (n, %)	3(5.00%)	2(6.67%)	2(6.67%)	0.727

For normal distribution, differences among three groups were analyzed using one-way ANOVA test. For non-normal distribution off data, differences among three groups were analyzed using Kruskal-Wallis test.

Values are expressed as the mean±S.D, median with interquartile range, or percentages.

PTMC: Papillary thyroid microcarcinoma

HT: Hashimoto’s thyroiditis

*Non-normal distribution of continuous variables.

P-value: The p-values were adjusted for age and sex for the trend.
