# Supplementary material for: FAM172A promotes follicular thyroid carcinogenesis and may be a marker of FTC
Source: Endocr Relat Cancer. 2020 Sep 21;27(11):657–69. doi: 10.1530/ERC-20-0181 (PMC7707803; doi:10.1530/ERC-20-0181)
Supplement: Table S2 The IHC staining scores of FAM172A from FFPEs samples [file supplementary_table_2.pdf]

Table S2 The IHC staining scores of FAM172A from FFPEs samples

	FAM172A				FAM172A		
Case#	SP	SI	SP×SI	Case#	SP	SI	SP×SI
FTC01	3	3	9	FT-UMP01	3	3	9
FTC02	4	3	12	FT-UMP02	3	3	9
FTC03	3	3	9	FT-UMP03	3	2	6
FTC04	2	2	4	FT-UMP04	2	1	2
FTC05	1	2	2	FT-UMP05	4	3	12
FTC06	0	0	0	FT-UMP06	2	2	4
FTC07	2	3	6	FT-UMP07	2	1	2
FTC08	4	3	12	FT-UMP08	3	1	3
FTC09	2	2	4	FT-UMP09	3	2	6
FTC10	2	2	4	FT-UMP10	3	2	6
FTC11	3	2	6	FT-UMP11	1	1	1
FTC12	3	3	9	FT-UMP12	1	2	2
FTC13	2	2	4	FT-UMP13	2	3	6
FTC14	1	3	3	FT-UMP14	3	2	6
FTC15	3	3	9	FT-UMP15	1	2	2
FTC16	2	3	6	FT-UMP16	2	2	4
FTC17	2	3	6	FT-UMP17	2	3	6
FTC18	3	2	6	FT-UMP18	3	2	6
FTC19	3	2	6	FT-UMP19	2	1	2
FTC20	2	2	4	FT-UMP20	1	2	2
FTC21	2	2	4	FT-UMP21	1	2	2
FTC22	2	3	6	FT-UMP22	2	1	2
FTC23	2	3	6	FT-UMP23	4	3	12
FTC24	2	2	4	FT-UMP24	1	1	1
FTC25	3	2	6	FT-UMP25	1	2	2
FTC26	4	3	12	FT-UMP26	1	1	1
FTC27	3	3	9	FT-UMP27	3	2	6
FTC28	2	2	4	FT-UMP28	2	2	4
FTC29	3	3	9	FT-UMP29	2	1	2
FTC30	4	3	12	FT-UMP30	3	2	6
FTC31	2	2	4	FTA01	0	0	0

FTC32	3	2	6	FTA02	2	1	2
FTC33	4	3	12	FTA03	2	3	6
FTC34	3	2	6	FTA04	2	1	2
FTC35	4	3	12	FTA05	1	1	1
FTC36	3	2	6	FTA06	1	1	1
FTC37	3	3	9	FTA07	0	0	0
FTC38	3	3	9	FTA08	3	2	6
FTC39	2	2	4	FTA09	2	1	2
FTC40	3	2	6	FTA10	1	2	2
FTC41	3	3	9	FTA11	2	3	6
FTC42	3	3	9	FTA12	2	1	2
FTC43	2	3	6	FTA13	2	1	2
FTC44	4	3	12	FTA14	1	2	2
FTC45	2	3	6	FTA15	2	2	4
FTC46	3	3	9	FTA16	2	1	2
FTC47	0	0	0	FTA17	1	2	2
FTC48	2	2	4	FTA18	2	1	0
FTC49	4	2	8	FTA19	2	1	2
FTC50	2	2	4	FTA20	0	0	0
FTC51	2	3	6	FTA21	2	1	2
FTC52	2	4	8	FTA22	0	0	0
FTC53	2	4	8	FTA23	2	3	6
FTC54	0	0	0	FTA24	0	0	0
FTC55	4	3	12	FTA25	2	1	2
FTC56	3	3	9	FTA26	2	3	6
FTC57	2	4	8	FTA27	2	1	2
FTC58	4	3	12	FTA28	1	2	2
FTC59	4	3	12	FTA29	3	1	3
FTC60	3	3	9	FTA30	1	1	1

Note: FTC, Follicular thyroid carcinoma; FT-UMP, Follicular tumor of uncertain malignant potential; FTA, Follicular thyroid adenoma. SP, Score of staining percentage; SI, Score of staining intensity;

SP scores: 0, no positive staining or < 5%; 1, 5%–25% positive; 2, 26%–50% positive; 3, 51%–75% positive; 4, 76%–100% positive. SI scores: 0, no staining; 1, weak; 2, moderate; 3, strong stain.
