# Supplementary material for: FAM172A promotes follicular thyroid carcinogenesis and may be a marker of FTC
Source: Endocr Relat Cancer. 2020 Sep 21;27(11):657–69. doi: 10.1530/ERC-20-0181 (PMC7707803; doi:10.1530/ERC-20-0181)
Supplement: Table S3 Clinical and pathological characteristics in 81 FNAB samples [file supplementary_table_3.pdf]

Table S3 Clinical and pathological characteristics in 81 FNAB samples

Clinicopathological variables	FTC (12)	FT-UMP (24)	FTA (45)	P value
Age (years)	54±13	47±10	51±14	0.307
<55 (n, %)	5(41.67%)	18(75.00%)	28(62.22%)	0.675
Male sex (n, %)	2(16.67%)	6(25.00%)	11(24.44%)	0.839
*Time of diagnosis (month)	1(1-9)	4(2-30)	1(1-49)	0.742
*FT3 (pmol/L)	5.14(4.60-5.36)	4.96(4.53-5.30)	4.64(4.20-4.87)	0.675
*FT4 (pmol/L)	18.44(15.06-20.15)	16.16(14.31-18.97)	18.03(15.55-18.47)	0.595
*TSH (mIU/l)	1.91(1.55-4.38)	1.61(0.79-2.02)	0.90(0.79-2.02)	0.183
*TG (ng/ml)	157.40(3.90-604.90)	62.79(14.91-264.23)	33.44(8.71-68.47)	0.046
*TGAb (KIU/L)	14.51(10.82-16.46)	13.87(11.68-33.70)	12.78(10.92-34.45)	0.275
*TPOAb (KIU/L)	9.06(6.55-11.44)	22.63(9.76-92.32)	6.33(6.08-167.23)	0.685
Single nodule (n, %)	10(83.33%)	18(75.00%)	28(62.22%)	0.008
*Tumor maximum diameter (cm)	2.0(1.6-5.0)	3.5(1.8-4.3)	2.0(1.8-3.0)	0.000
Minimally invasive (n, %)	7(58.33%)	-	-	-
Angioinvasion (n, %)	3(25.00%)	-	-	-
Lymph node metastasis (n, %)	3(25.00%)	-	-	-
Distant metastases (n, %)	2(16.67%)	-	-	-
Extrathyroidal invasion (n, %)	3(25.00%)	-	-	-
Compared with PTMC (n, %)	2(16.67%)	3(12.50%)	7(15.56%)	0.928
Compared with HT (n, %)	1(8.33%)	7(29.17%)	5(11.11%)	0.113

For normal distribution, differences among three groups were analyzed using one-way ANOVA test. For non-normal distribution off data, differences among three groups were analyzed using Kruskal-Wallis test.

Values are expressed as the mean±S.D, median with interquartile range, or percentages.

PTMC: Papillary thyroid microcarcinoma

HT: Hashimoto’s thyroiditis

*Non-normal distribution of continuous variables.

P-value: The p-values were adjusted for age and sex for the trend.
