# Supplementary material for: FAM172A promotes follicular thyroid carcinogenesis and may be a marker of FTC
Source: Endocr Relat Cancer. 2020 Sep 21;27(11):657–69. doi: 10.1530/ERC-20-0181 (PMC7707803; doi:10.1530/ERC-20-0181)
Supplement: Table S2 The IHC staining scores of FAM172A from FNAB samples [file supplementary_table_4.pdf]

**Table S2 The IHC staining scores of FAM172A from FNAB samples**

| | FAM172A | | | | FAM172A | | |
|---|---|---|---|---|---|---|---|
| Case# | SP | SI | SP×SI | Case# | SP | SI | SP×SI |
| Case 01 | 0 | 0 | 0 | Case 42 | 2 | 1 | 2 |
| Case 02 | 3 | 2 | 6 | Case 43 | 3 | 3 | 9 |
| Case 03 | 0 | 0 | 0 | Case 44 | 0 | 1 | 0 |
| Case 04 | 2 | 1 | 2 | Case 45 | 1 | 2 | 2 |
| Case 05 | 1 | 1 | 1 | Case 46 | 2 | 1 | 2 |
| Case 06 | 4 | 3 | 12 | Case 47 | 3 | 2 | 6 |
| Case 07 | 3 | 2 | 6 | Case 47 | 3 | 2 | 6 |
| Case 08 | 0 | 1 | 0 | Case 49 | 3 | 1 | 3 |
| Case 09 | 1 | 3 | 3 | Case 50 | 1 | 1 | 1 |
| Case 10 | 0 | 0 | 0 | Case 51 | 2 | 3 | 6 |
| Case 11 | 3 | 3 | 9 | Case 52 | 1 | 1 | 1 |
| Case 12 | 1 | 2 | 2 | Case 53 | 2 | 3 | 6 |
| Case 13 | 0 | 0 | 0 | Case 54 | 2 | 1 | 2 |
| Case 14 | 1 | 2 | 2 | Case 55 | 0 | 0 | 0 |
| Case 15 | 2 | 2 | 4 | Case 56 | 0 | 1 | 0 |
| Case 16 | 1 | 1 | 1 | Case 57 | 4 | 2 | 8 |
| Case 17 | 0 | 1 | 0 | Case 58 | 0 | 1 | 0 |
| Case 18 | 4 | 3 | 12 | Case 59 | 3 | 1 | 3 |
| Case 19 | 2 | 1 | 2 | Case 60 | 4 | 2 | 8 |
| Case 20 | 0 | 1 | 0 | Case 61 | 1 | 2 | 2 |
| Case 21 | 1 | 2 | 2 | Case 62 | 3 | 3 | 9 |
| Case 22 | 0 | 0 | 0 | Case 63 | 0 | 1 | 0 |
| Case 23 | 2 | 1 | 2 | Case 64 | 1 | 2 | 2 |
| Case 24 | 1 | 1 | 1 | Case 65 | 2 | 1 | 2 |
| Case 25 | 4 | 3 | 12 | Case 66 | 3 | 2 | 6 |
| Case 26 | 2 | 1 | 2 | Case 67 | 0 | 0 | 0 |
| Case 27 | 3 | 2 | 6 | Case 68 | 4 | 3 | 12 |
| Case 28 | 2 | 1 | 2 | Case 69 | 2 | 3 | 6 |
| Case 29 | 0 | 0 | 0 | Case 70 | 2 | 1 | 2 |
| Case 30 | 3 | 3 | 9 | Case 71 | 1 | 1 | 1 |
| Case 31 | 1 | 2 | 2 | Case 72 | 2 | 3 | 6 |

| | | | | | | | |
|---|---|---|---|---|---|---|---|
| Case 32 | 3 | 2 | 6 | Case 73 | 2 | 1 | 2 |
| Case 33 | 0 | 0 | 0 | Case 74 | 0 | 0 | 0 |
| Case 34 | 2 | 2 | 4 | Case 75 | 0 | 1 | 0 |
| Case 35 | 1 | 1 | 1 | Case 76 | 0 | 1 | 0 |
| Case 36 | 0 | 1 | 0 | Case 77 | 3 | 1 | 3 |
| Case 37 | 0 | 0 | 0 | Case 78 | 0 | 0 | 0 |
| Case 38 | 2 | 1 | 2 | Case 79 | 1 | 2 | 2 |
| Case 39 | 0 | 1 | 0 | Case 80 | 2 | 1 | 2 |
| Case 40 | 3 | 2 | 6 | Case 81 | 0 | 0 | 0 |
| Case 41 | 0 | 0 | 0 | | | | |

Note: FTC, Follicular thyroid carcinoma; FT-UMP, Follicular tumor of uncertain malignant potential; FTA, Follicular thyroid adenoma. SP, Score of staining percentage; SI, Score of staining intensity.

SP scores: 0, no positive staining or < 5%; 1, 5%–25% positive; 2, 26%–50% positive; 3, 51%–75% positive; 4, 76%–100% positive.SI scores: 0, no staining; 1, weak; 2, moderate; 3, strong stain.
